# Data driven water quality assessment using machine learning and synthetic data generation

**DOI:** 10.1038/s41598-026-54560-7

**Published:** 2026-06-05

**Authors:** Naga Durga Satya Siva Kiran Relangi, D. Venkata Naga Raju, P. Venkata Rama Raju, M. Vishnu Vardhana Rao, Krishna Kant Pandey, Vinod Yadav

**Affiliations:** 1 Department of Information Technology, Shri Vishnu Engineering College for Women (Autonomous), Bhimavaram, India; 2 Department of Computer Science and Engineering (DS), Vignan’s Institute of Management & Technology for Women, Hyderabad, India; 3 Manipal University Jaipur, Jaipur, India

**Keywords:** Logistic regression (LR), Extreme gradient boosting (XGB), Gradient boosting (GB), Random forest (RF), Synthetic minority oversampling technique (SMOTE), Water quality index (WQI), Scientific data, Software

## Abstract

Providing fresh and clean water to all is the objective of Sustainable Development Goal 6. Consuming clean water improves health for all living beings worldwide. Therefore, accurately estimating the water quality and classifying the type of water (i.e. whether it is used for potable purposes or not) based on physicochemical parameters is considered a challenging issue due to the dynamic nature of water quality data, the selection of physical or data-driven models used to estimate the water quality and the lack of a large water dataset for training the data-driven models. The primary objective of this research is to generate synthetic dataset(s) and propose state-of-the-art machine learning models for predicting water quality. In this study, synthetic data generation via oversampling is used to balance the water quality dataset and train models on a balanced dataset for water quality classification and prediction. The experimental results indicate that the quality of the synthetic Drinking Water Final dataset produced using SMOTE is satisfactory, as evidenced by a Maximum Mean Discrepancy (MMD) score of 0.0067. Furthermore, the test accuracy of the GB and XGB machine learning models is notably high at 99.47% on the synthetic Drinking Water Final dataset. The performance of the ML model on the synthetic Drinking Water Final dataset generated by a GAN is outstanding; however, the MMD score indicates that the quality of the synthetic data is subpar. Likewise, the MMD score quality of the synthetic Water Quality Analysis dataset produced using SMOTE (MMD = 0.0006) and GAN (MMD = 0.0016) is noteworthy. The comprehensive evaluation findings demonstrate that the synthetic dataset produced through oversampling techniques will enhance the models’ predictive accuracy.

**Supplementary Information:**

The online version contains supplementary material available at 10.1038/s41598-026-54560-7.

## Introduction

All life on Earth is contingent upon natural resources. Individuals are obligated to conserve and preserve natural resources safely and in an environmentally friendly manner. Nonetheless, it has become increasingly essential to assess the quality of these resources due to several factors, including pollution, industrial activities, waste disposal sites, and other forms of contamination. In light of escalating environmental concerns, environmental monitoring and management have garnered significant attention. Therefore, it is essential to evaluate the quality of natural resources. Assessments of water excellence and its adequacy for various species are conducted by Jude, P. Sebastian Vindro et al.^1^. Water contamination diminishes water quality, adversely affecting the health of marine organisms and, consequently, humans who consume them. Jain et al.^2^. Management of groundwater quality is crucial for sustainable development, Kiran RNDSS et al.^3^. Traditional techniques for evaluating groundwater quality primarily rely on mathematical modelling. These models are not universally applicable due to their significant data requirements, high computational demands, and the assumption of a linear relationship between the dependent and independent variables Kiran RNDSS et al.^4^. Water quality index (WQI) is one conventional method to assess the total water quality by integrating physical, chemical, and biological characteristics R Kiran Relangi, N.D.S.S et al.^5^, Shiri N et al.^6^. It streamlines the evaluation of water quality as a composite metric^7–9^.

The evaluation of water quality presents numerous challenges, including the time-consuming and expensive processes of large-scale sample collection, testing, and data management^10–14^. Synthetic data generation is one approach to addressing the scarcity of water quality data. Furthermore, upon examining the prior literature, the authors are aware of very few studies that have investigated and validated the efficacy of a machine learning model on an oversampled dataset for forecasting groundwater quality.

The objective of this work is to evaluate water quality using three water-quality datasets and state-of-the-art machine-learning models. The objectives of the study are as follows:

- The three datasets are acquired from the public repositories.
- After acquiring the datasets, implement the four machine learning models (RFC, LR, GB and XGB) on the datasets (imbalanced).
- Make the datasets balanced using over-sampling techniques (SMOTE, MAHAKIL and GAN).
- The quality of the synthetic data generated (using SMOTE, MAHAKIL and GAN) is evaluated using visual overlap, correlation heatmap and distance metrics.
- Once the three datasets are balanced, the four ML models are applied to identify the best predictive water quality model by comparing them using the Receiver Operating Characteristic Area Under the Curve(ROC-AUC) curve and classifier metrics.

The remainder of the research article is organised as follows: Section “Literature survey” provides an overview of relevant research and studies by various scholars on artificial intelligence approaches for water quality prediction and categorisation. The mechanism used to classify and predict water quality is described in Section “Methodology” of the article. The comparison study and analysis of the experimental results were covered in full in Section “Results and discussion”. The conclusion and upcoming projects are presented in the final section.

## Literature survey

In recent years, artificial intelligence (AI) and its sub-branches, such as machine learning (ML) approaches, have been widely employed to analyse surface and groundwater quality, including WQI calculation^15–18^. These techniques have proven effective tools for simulating complex nonlinear phenomena in water resource studies. The following sub-section demonstrates the applicability of machine learning models in hydrology.

Water Quality Classification and Prediction employ AI and ML to forecast the water quality class, serving as a well-established approach to streamline the computation of water quality evaluations Tung, T. M., Yaseen, Z. M.^19^. Sakizadeh M et al.^20^ evaluated three ANN algorithms: early stopping, an ensemble of ANNs, and Bayesian regularisation. Among these, Bayesian regularisation excelled, achieving the lowest MSE (MSE) of 7.71, which reflects its superior generalisation capability and a lower tendency to overfit compared to the ensemble method (MSE = 9.25) and early stopping. The successful application of these ANN algorithms, especially Bayesian regularisation, can significantly improve the accuracy of WQI predictions.

Kadam AK et al.^21^ employed the Levenberg–Marquardt three-layer backpropagation technique to develop an ANN model for forecasting the WQI of groundwater in the Shivganga River basin, recognized for its efficacy in training and error reduction. Multiple Linear Regression (MLR) was utilised for comparative performance analysis. The ANN model demonstrated reliable and consistent estimates of groundwater quality in both seasons. The ANN model outperformed the MLR model in accuracy, error reduction, and reliability for forecasting groundwater quality. Gazzaz NM et al.^22^ employed various essential algorithms to construct a reliable model for forecasting the WQI, including 1. Artificial Neural Network (ANN) 2. The Quick Propagation (QP) Algorithm enhances the training of artificial neural networks (ANNs) by ensuring rapid convergence and stability, surpassing conventional batch backpropagation techniques.

This study examines the forecasting of groundwater quality in the Amol-Babol aquifer using an ANN model, with a particular focus on sodium (Na) concentration due to its effects on irrigation (Heidarzadeh N.^23^). The ANN model achieved high accuracy, with R^2^ values of 0.99 for training and 0.98 for validation and a root mean square error (RMSE) of 0.08. The results indicate that the ANN model is an efficient and economical approach for forecasting groundwater quality. Sahu M, Mahapatra SS, et al.^24^ reported that conventional approaches to water quality evaluation frequently encounter the uncertainty and inaccuracy inherent in environmental data. The paper proposes an ANFIS model that integrates the principles of fuzzy logic and neural networks. The study suggests that ANFIS can accurately forecast the WQI, which is crucial for effective water quality management.

The researchers Hosseini-Moghari S-M et al.^25^ state that WQIs are commonly employed tools for assessing water quality status. Nevertheless, the utilisation of these indices is constrained by factors such as a lack of requisite databases or ambiguity in decision-making. In the course of the research, FWQIs were formulated utilising the Mamdani fuzzy inference system (FIS) to address the shortcomings mentioned above. The purpose of this study was to compare the water quality analysis capabilities of the WQI and the Fuzzy Inference System (FIS)–based WQI. The findings indicated that knowledge-based models, such as FIS, were effective and adaptable instruments for integrating expert opinions and modelling the uncertainties associated with water supplies and environmental complexities. It has been noted that FWQI is a more practical and effective tool for assessing water quality.

Pradeep Kumar GN et al.^26^ examined 14 training algorithms for training feed-forward neural networks for groundwater level forecasting. The Levenberg–Marquardt algorithm was found to be particularly efficient and accurate (R2 = 0.966) for predicting groundwater levels. Saghebian et al.^27^ conducted research to establish a decision-tree-based methodology for classifying groundwater quality in the Ardebil region of Iran, using data from agricultural areas. The researchers utilised principal component analysis (PCA) in conjunction with the decision tree classifier to determine appropriate criteria for classifying groundwater quality. The model attained a correctly classified instances (CCI) of 0.88 and a kappa statistic of 0.83, signifying excellent accuracy and robust classification agreement.

Compared with other traditional modelling approaches, Najah A, El-Shafie A et al.^28^ found that the ANN is a novel method with a flexible mathematical framework that can detect intricate non-linear correlations between input and output data. Three distinct model approaches were used in this investigation: LRM, multilayer perceptron neural networks (MLP-NN), and RBF-NN. Of the three models, the RBF-NN requires less computational power to train and converges to a solution more quickly than the MLP-NN. Furthermore, with minimal processing, the RBF-NN improved the accuracy of water-quality parameter prediction.

Recent advancements in machine learning (ML) methodologies have empowered researchers to efficiently process and analyse large datasets, meeting the rigorous demands of comprehensive water quality assessments. ML, a subset of AI, utilises algorithms to analyse data and identify patterns to predict new information. Advanced data analysis and processing techniques are extensively employed across many fields owing to their high precision, adaptable customisation, and straightforward extensibility. Chen et al.^29^ evaluated various machine-learning models for simulating groundwater dynamics, demonstrating their superiority. These results substantiate the need to use these strategies. Chen et al. recommended the utilisation of cost-sensitive learning models and ensemble learning models for predicting drinking water quality. Islam Khan, MdS et al.^30^ utilised PCA and a Gradient Boosting Classifier for water quality classification and prediction, and the results indicated that the Gradient Boosting Classifier classified water quality status more efficiently.

Raheja H et al.^31^ evaluated the performance of three state-of-the-art machine learning models, Extreme gradient boosting (XGBoost), Gradient boosting machine (GBM), and Deep neural network (DNN) for water quality and prediction and the results indicate the DNN model outperformed the other two models. Senapati C et al.^32^ proposed a machine learning-based solution for forecasting and monitoring groundwater quality in Telangana, which is demonstrated using a stacking ensemble method. The suggested method integrates decision trees (DT) with meta-models, such as LR, K-NN, BRR, and random forest. The stacking ensemble model outperformed the decision tree model (accuracy = 96.07%).

Tabassum S et al.^33^ studied the performance of ML models for water quality index prediction, and their findings demonstrate that ML-based models are competent for this task. In a similar study conducted by Kuruvilla E and Kundapura S^34^, the results indicate that machine learning models are accurate and reliable for predicting groundwater potability. Singh RI and Lilhore UK^35^ proposed a hybrid classification method using PAC, K-means, and a voting classifier. The hybrid model effectively separates the potable and non-potable classes with approximately 96% accuracy. Similarly, Yurtsever & Murat^36^, Domakonda VK^37^, Nitharshni et al.^38^, and Khoi DN^39^ employed the ML models to classify the water quality and accurately predict the WQI. Researchers Ho JY et al.^40^ applied the ID3 tree to predict the WQI for the Klang River and classify it within a specific water quality class. Nguyen PT et al.^41^ proposed ensemble soft-computing models for groundwater potential mapping in Dak Lak Province, Vietnam. Developed a network architecture for assessing the quality of the water for drinking and irrigation purposes. The authors Chen S et al.^29^ enhanced the BP network through the grey correlation analysis method to forecast water quality. Asadollah SBHS et al.^42^ applied extra trees to predict WQI values in the Tsuen River, Hong Kong.

Overall, ML approaches have been extensively used in water quality classification prediction^43,44^. To improve prediction performance, the studies used MLP, RF, SVM, KNN, Naive Bayes, Gradient Boosting, XGB, various ensemble learning models, and cost-sensitive learning models. These techniques require further research and tuning to account for additional factors and larger datasets, thereby increasing model predictive power. Table 1 presents existing studies that applied k-fold validation to make their models robust.

**Table 1:** Summary of existing approaches with cross-validation technique.

Study	Dataset/samples	Models used	Key findings/best accuracy	Oversampling used	k-Fold CV
^27^	750 samples from 73 wells over 15 years	Decision tree	88.13% accuracy, κ = 0.833	No	tenfold
^29^	Multi-station physicochemical dataset	ANN, SVM, DT, RF	RF best (> 95% accuracy)	No	tenfold
^30^	River basin dataset (hundreds of samples)	RF, SVM, ANN, KNN, DT	RF best (95–98%)	No	tenfold
^31^	392 groundwater samples (Haryana, India)	DNN, XGBoost, GBM	DNN best (CC = 0.989 EWQI)	No	fivefold

### Research gap

In conclusion, the literature above demonstrates that machine learning-based water quality prediction models are dependable and effective. The current advancements in water quality modelling exhibit the following research deficiencies:

1. To check the quality of the water, it needs to be monitored all the time and in great detail. Testing all the water quality parameters in a lab is quite expensive. So, it costs a lot to collect many water samples.
2. Even though samples are collected on a large scale, the exploratory data analysis reveals that the dataset is unbalanced. It necessitates the utilisation of synthetic data production.
3. From the existing literature, it is noticed that most of the studies train the machine learning models with small and imbalanced datasets.
4. If training the machine learning models with a smaller number of samples may trigger overfitting.
5. The researchers have not evaluated the quality of synthetic data; instead, they rely on the downstream classifier accuracy metrics only.

This research gap motivates us to use synthetic data generation methods to balance the dataset and train state-of-the-art machine learning models on the balanced dataset to improve the accuracy of the water quality prediction task.

## Methodology

**Fig. 1:**
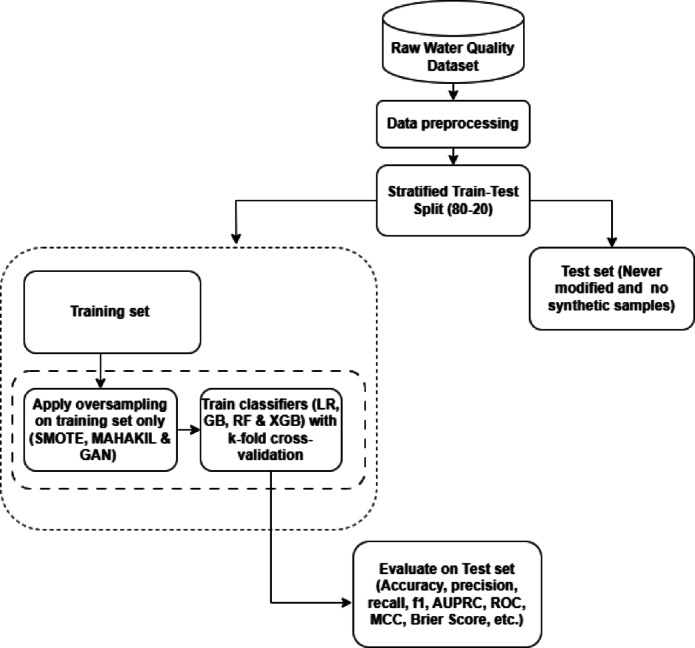
Methodology adopted for water quality classification and prediction.

The primary goal of this research is to predict water quality with high precision, thereby alerting the general public and water management stations to maintain it. The methodology of this study is shown in Fig. 1. It begins with preprocessing the water quality datasets using min–max normalisation. The mathematical formula of min–max normalisation is given in Eq. (1). The dataset is divided into training and validation subsets using an 80–20 ratio. It ensures a fair representation of each class and prevents bias during model training and evaluation. To ensure robust model evaluation, k-fold cross-validation is employed on the training dataset^45^. Furthermore, to address class imbalance, oversampling techniques are exclusively applied to the training dataset to prevent data leakage. This is followed by selecting a machine learning model based on an analysis of various models’ performance to identify the most accurate predictor of water quality. This step involves adopting various training strategies, addressing class imbalance through synthetic data generation, and evaluating the model’s performance in predicting water quality across diverse datasets. In the next step, the results of each model are analysed in terms of accuracy, precision, recall, F1-score, Brier Score and other metrics. Sensitivity analysis of the models was also performed using ROC curves.

1 $$x_{scaled} = \frac{{x_{i} - x_{min} }}{{x_{max} - x_{min} }}$$

In the above Eq. (1), the parameter $x_{i}$ is the current value to be transformed, $x_{\min }$ and $x_{\max }$ These are the smallest and largest values of the feature, respectively.

### Dataset description

In this study, we utilised three datasets from well-known repositories to comprehensively cover water quality, as listed in Table 2. Datasets were considered based on their applicability, quality, and information coverage. Descriptions of the used datasets are given below.

**Table 2:** Water quality data sets information.

Name of the dataset	Total features	Total samples	No. of potable samples	No. of not potable samples
Drinking water final dataset^46^	13	718	441	277
Water potability dataset^47^	10	3276	1278	1998
Water quality analysis dataset^48^	21	7999	912	7087

#### Drinking water final dataset

Table 3 presents the features and descriptive statistics for each feature in the Drinking Water Final Dataset. The dataset comprises 781 samples. The dataset is sourced from IEEE DataPort. The parameters WQI and Potable state the condition of the water.

**Table 3:** Drinking water final dataset statistical information.

Feature	Type	Missing value count	mean	std	min	25%	50%	75%	max
pH	Numeric	0	7.6773	0.6889	2.41	7.27	7.64	8.11	10.01
Sodium	Numeric	0	84.891	147.32	0	19.053	38.525	70.48	1158
Magnesium	Numeric	0	56.268	113.8	0	14	19	36.83	990
Calcium	Numeric	0	70.927	141.33	0	18.075	28.85	62	1158
Chloride	Numeric	0	119.84	344.77	0	17.75	36.24	76.6	4700
Potassium	Numeric	0	12.113	17.271	0	3.8	6.935	11	110
Carbonate	Numeric	0	117.96	93.635	0	54.613	116.16	145	580.7
Sulphate	Numeric	0	50.705	78.489	0	14.4	35.04	53.89	1159
TDS	Numeric	0	499.61	978.48	0	0	195.75	696.5	11,619
EC	Numeric	0	1012.4	1465.5	2.1	434.25	543.4	1020	16,598
TH	Numeric	0	197.82	154.2	0	116.25	162	271.5	1400
WQI	Numeric	0	54.36	16.59	13.23	42.655	57.866	65.82	89.22
Potable	Binary	0	0.3245	0.4685	0	0	0	1	1

#### Water potability dataset

Table 4 presents the features and descriptive statistics of each feature present in the Water Potability Dataset. The dataset comprises 3275 samples. To standardise the dataset, we applied min–max normalisation. Data imputation (mean imputation) is used to fill the missing values for the Sulfate and Trihalomethanes attributes. The potable feature in the dataset is used as the class label to distinguish between potable and non-potable water cases.

**Table 4:** Water potability dataset statistical information.

Feature	Type	Missing value count	mean	std	min	25%	50%	75%	max
ph	Numeric	491	6.02137	2.92276	0	5.28445	6.73700	7.87031	14
Hardness	Numeric	0	196.366	32.8844	47.432	176.847	196.952	216.669	323.124
Solids	Numeric	0	22,014.47	8769.88	320.942	15,665.1	20,933.5	27,334.1	61,227.2
Chloramines	Numeric	0	7.12222	1.58332	0.352	6.12703	7.13016	8.11504	13.127
Sulfate	Numeric	781	284.931	94.4606	129	240.548	318.643	350.372	481.030
Conductivity	Numeric	0	426.162	80.8003	181.48	365.729	421.879	481.754	753.342
Organic_carbon	Numeric	0	14.2861	3.30796	2.2	12.0660	14.2193	16.5581	28.3
Trihalomethanes	Numeric	162	63.1421	21.2451	0.738	53.7876	65.4445	76.6515	124
Turbidity	Numeric	0	3.96709	0.78030	1.45	3.4400	3.95509	4.50043	6.739
Potability	Binary	0	0.39022	0.4878	0	0	0	1	1

#### Water quality analysis dataset

Table 5 presents the features and descriptive statistics of each feature present in the Water Quality Analysis Dataset. The dataset comprises 7999 samples. The Water Quality Analysis dataset includes the target class label, i.e., potable. To standardise the dataset, we applied min–max normalisation.

**Table 5:** Water quality analysis data sets statistical information.

Feature	Type	Missing value count	mean	std	min	25%	50%	75%	max
aluminium	Numeric	0	0.666158	1.265145	0	0.04	0.07	0.28	5.05
ammonia	Numeric	0	14.27627	8.878109	− 0.08	6.575	14.13	22.13	29.84
arsenic	Numeric	0	0.161445	0.25259	0	0.03	0.05	0.1	1.05
barium	Numeric	0	1.567715	1.216091	0	0.56	1.19	2.48	4.94
cadmium	Numeric	0	0.042806	0.036049	0	0.008	0.04	0.07	0.13
chloramine	Numeric	0	2.176831	2.567027	0	0.1	0.53	4.24	8.68
chromium	Numeric	0	0.247226	0.27064	0	0.05	0.09	0.44	0.9
copper	Numeric	0	0.805857	0.653539	0	0.09	0.75	1.39	2
flouride	Numeric	0	0.771565	0.435373	0	0.405	0.77	1.16	1.5
bacteria	Numeric	0	0.319665	0.329485	0	0	0.22	0.61	1
viruses	Numeric	0	0.328583	0.378096	0	0.002	0.008	0.7	1
lead	Numeric	0	0.09945	0.058172	0	0.048	0.102	0.151	0.2
nitrates	Numeric	0	9.818822	5.541331	0	5	9.93	14.61	19.83
nitrites	Numeric	0	1.329961	0.573219	0	1	1.42	1.76	2.93
mercury	Numeric	0	0.005194	0.002967	0	0.003	0.005	0.008	0.01
perchlorate	Numeric	0	16.4603	17.68747	0	2.17	7.74	29.48	60.01
radium	Numeric	0	2.920548	2.323009	0	0.82	2.41	4.67	7.99
selenium	Numeric	0	0.049685	0.02877	0	0.02	0.05	0.07	0.1
silver	Numeric	0	0.147781	0.143551	0	0.04	0.08	0.24	0.5
uranium	Numeric	0	0.044673	0.026904	0	0.02	0.05	0.07	0.09

**Fig. 2:**
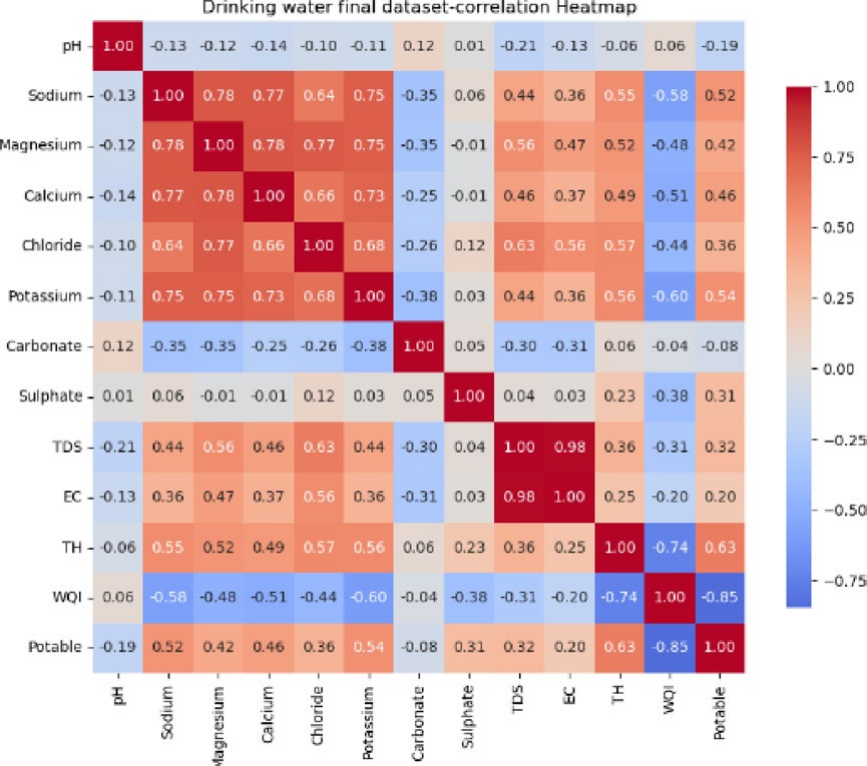
Correlation heatmap for drinking water final dataset.

**Fig. 3:**
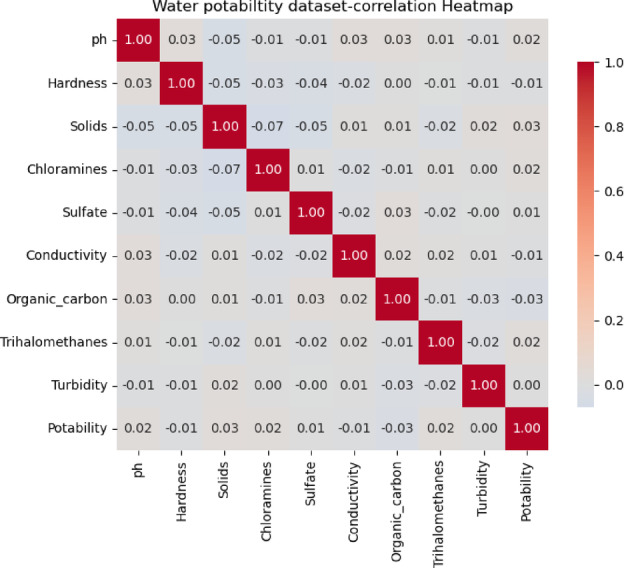
Correlation heatmap for waterpotability dataset.

**Fig. 4:**
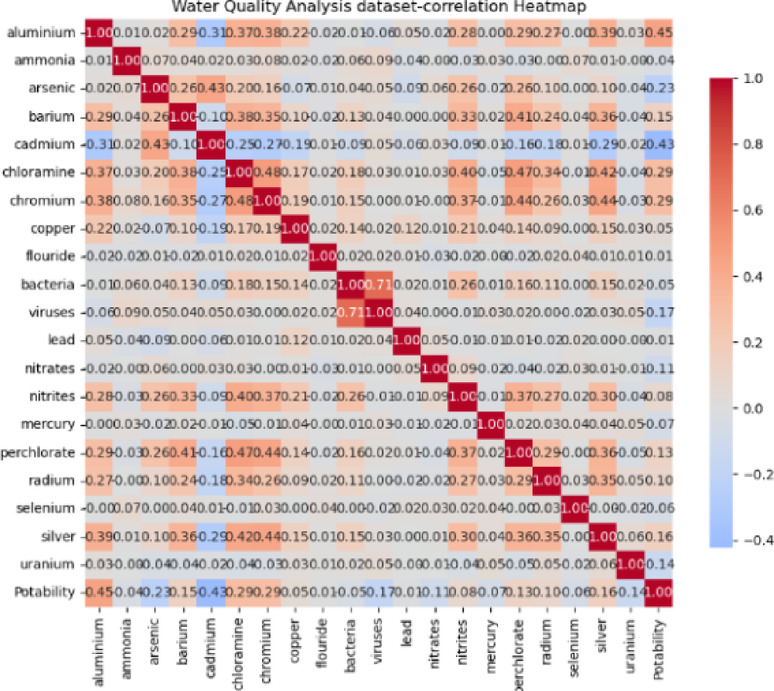
Correlation heatmap for water quality analysis dataset.

The correlations among the attributes in the water quality dataset(s) are analysed using a heatmap of the correlation matrix. The Heatmap is a visual representation of all the attribute values present in the dataset. Figure 2 depicts the correlation Heatmap of Drinking Water Final Dataset, Fig. 3 is the Heatmap of the Water Potability dataset, and Fig. 4 is the Heatmap of the Water Quality Analysis dataset.

### Classifiers for predicting the water quality

This subsection introduces the four ML models we adopted for water quality classification and prediction.

- Random Forest is one kind of ensemble learning approach it constructs various decision trees iteratively and combines their outputs to attain more accuracy and minimise or get around overfitting. Every tree is built on a different subset of the data, and the final prediction is typically made by taking the majority vote (for classification) or the average (for regression) of the individual trees. Minimise the classification error using Eq. 2 (for classification tasks) or minimise the mean squared error (MSE) using Eq. 3 (for regression tasks by averaging the predictions of several decision trees.
  2 $$Minimize\; Classificaiton\; Error = \frac{1}{{\mathrm{N}}}\sum\nolimits_{i = 1}^{N} {1\left( {\hat{y}_{i} \ne y_{i} } \right)}$$
  3 $$Minimize \;Mean \;Squared \left( {MSE} \right) = \frac{1}{{\mathrm{N}}}\sum\nolimits_{i = 1}^{N} {\left( {y_{i} - \hat{y}_{i} } \right)^{2} }$$
- A linear model for two-class classification is LR (LR). Logistic functions evaluate the probability that an input belongs to a class. The model aims to find the best-fitting line that separates the classes by maximising the likelihood of the observed data. Minimise the log-loss (or cross-entropy loss), which measures the difference between the estimated probabilities and the actual class labels using Eqs. 4–6.
  4 $$Minimize\;J\left( \theta \right) = \frac{1}{{\mathrm{N}}}\sum\nolimits_{i = 1}^{N} {\left[ {y_{i} \log \left( {\hat{p}_{i} } \right) + \left( {1 - y_{i} } \right)\log \left( {1 - \hat{p}_{i} } \right)} \right]}$$
  5 $$\hat{p}_{i} = \sigma \left( {\theta^{T} x_{i} } \right)$$
  6 $$\sigma \left( z \right) = \frac{1}{{1 + e^{ - z} }}$$
- GBM is an ensemble technique that constructs models iteratively, where each new model focuses on correcting the errors of the previous ones. It combines models with high error rates, i.e., weak learners, to form a robust predictor, gradually improving performance with each iteration. Minimise the loss function (e.g., log-loss for classification, MSE for regression) by sequentially adding models that correct the errors of the previous models using the following Eqs. 7 and 8.
  7 $$Minimize \;L\left( {y, \hat{y}} \right) = \sum\nolimits_{i = 1}^{N} {L\left( {y_{i} , \hat{y}_{i} } \right)}$$
  8 $$\hat{y}^{\left( t \right)} = \hat{y}^{{\left( {t - 1} \right)}} + \eta \cdot h_{t} \left( x \right)$$
- XGBoost is an advanced implementation of gradient boosting (gb) that is highly optimised for speed and performance. It includes additional features like regularisation (to reduce overfitting), parallel processing, and handling of missing values, making it one of the most powerful algorithms for structured data. Minimise a regularised loss function (Eqs. 9 and 10), which typically includes both the loss (e.g., log-loss, mse) and a regularisation term to prevent overfitting.
  9 $$Minimize \;Obj\left( \theta \right) = \sum\nolimits_{i = 1}^{N} {L\left( {y_{i} , \hat{y}_{i} } \right) } + \sum\nolimits_{t = 1}^{T} {\Omega \left( {f_{t} } \right)}$$
  10 $$\Omega \left( {f_{t} } \right) = \gamma T + \frac{1}{2} \lambda \sum\nolimits_{j = 1}^{J} {\omega_{j}^{2} }$$

The objective of this research is to propose a machine learning model for predicting water quality based on the physicochemical parameters in the dataset. In this work, we used 3 diverse datasets with different water quality parameters. The datasets are fine-tuned through preprocessing. The data samples have been partitioned into a training set (80%) and a test set (20%). While training the models, one essential aspect is to identify the hyperparameters.

### Data augmentation through synthetic data generation methods

The following subsection presents synthetic data generation methods to address class imbalance in the three water quality datasets, thereby creating balanced datasets and enhancing the predictive power of ML models.

- SMOTE

An imbalanced dataset is one in which the target class labels are not evenly distributed. Training the model using an imbalanced dataset will lead to poor performance. One of the predominant methods for handling imbalanced datasets is oversampling or undersampling. The Synthetic Minority Oversampling Technique (SMOTE) is a common method for oversampling minority classes by generating synthetic samples, which can lead to poor training results in unbalanced datasets. The root cause of this issue is ignoring the minority class, but the fact is that it is equally important as the majority class. To address this issue, we employ oversampling of the minority class in the unbalanced dataset.

SMOTE operates by locating neighbouring instances in the feature space, connecting them with a line, and producing a new sample at a point along that line. An example from the minority class is selected at random. Subsequently, k adjacent neighbours (often k = 5) are found, for instance, and a random neighbour is subsequently selected. A synthetic instance is created at a randomly selected location in the feature space between the two examples Patel J, Amipara C et al.^49^.

**Pseudocode:**
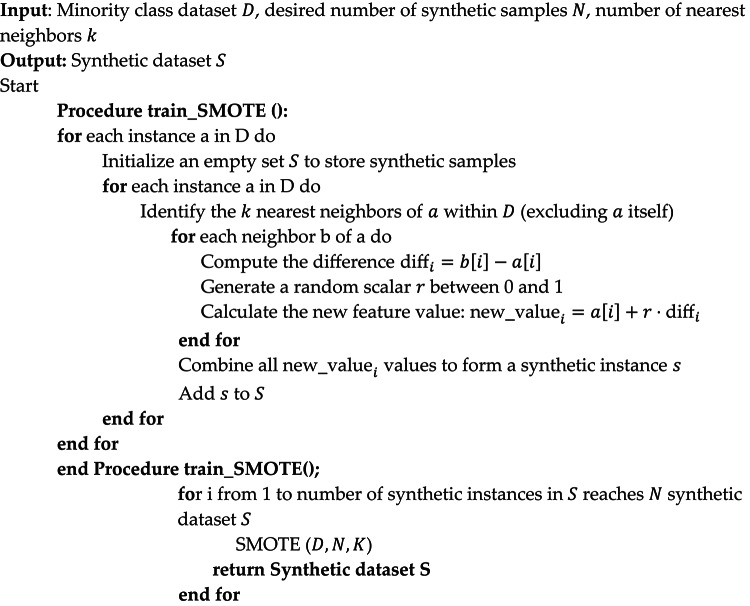
SMOTE.

(b) Mahakil oversampling approach for synthetic data generation

Inspired by biological studies, Bennin et al.^50^ proposed an oversampling method known as MAHAKIL. Initially, it computes the Mahalanobis distance between the mean vector and all other instances, then sorts them in ascending order by Mahalanobis distance. In the second stage, the data is partitioned into two segments, and synthetic data is generated based on principles of chromosomal inheritance. The average of two equally indexed samples from each partition is computed and regarded as synthetic data. The synthetic data produced by MAHAKIL exhibits greater diversity than SMOTE-based methods, effectively mitigating overgeneralization. The pseudocode for MAHAKIL is presented below.

**Pseudocode:**
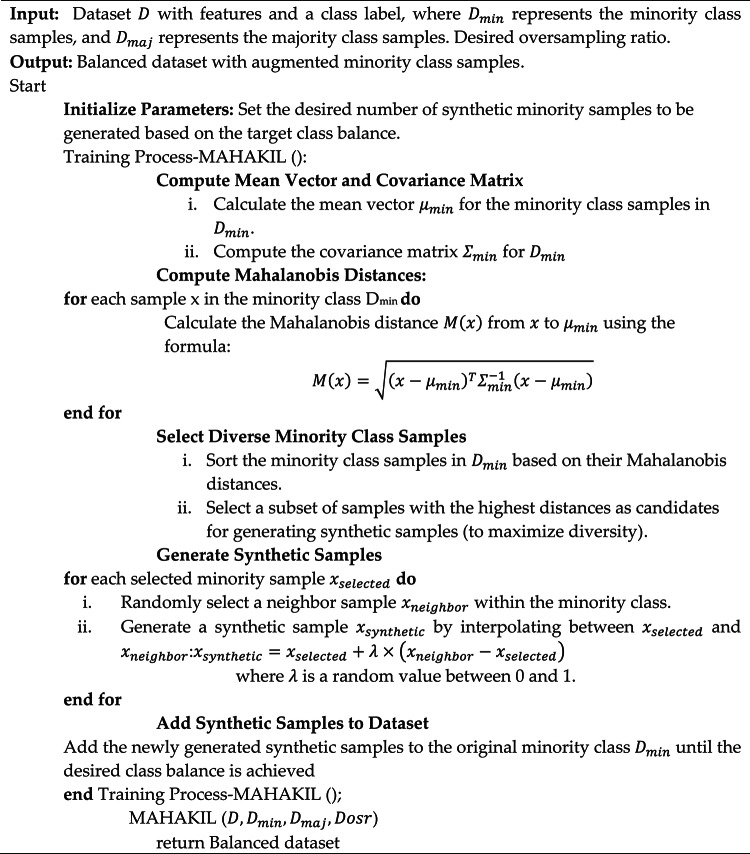
MAHAKIL (diversity-based oversampling approach).

The MAHAKIL-based oversampling method employs a stochastic linear interpolation strategy between minority and randomly selected majority samples, controlled by a uniformly distributed blending coefficient. The blending coefficient (α) is randomly drawn between 0 and 1 for each synthetic instance. The process iterates sequentially over minority samples and continues until the minority class is balanced with the majority class (Table 6).

**Table 6:** Hyperparameter values of the MAHAKIL.

Hyperparameter	Value
sampling_strategy	Balanced minority to majority
n_samples_needed	majority_count—minority_count
generatioin_method	Linear interpolation method (blending)
alpha (blending coefficient)	[0–1]
label_assignment	Minority class label
Stopping_criteria	Len(synthetic_samples) ≥ n_samples_needed
randomness_source	uniform random distribution

(c) Generative adversarial networks for synthetic data generation

**Fig. 5:**
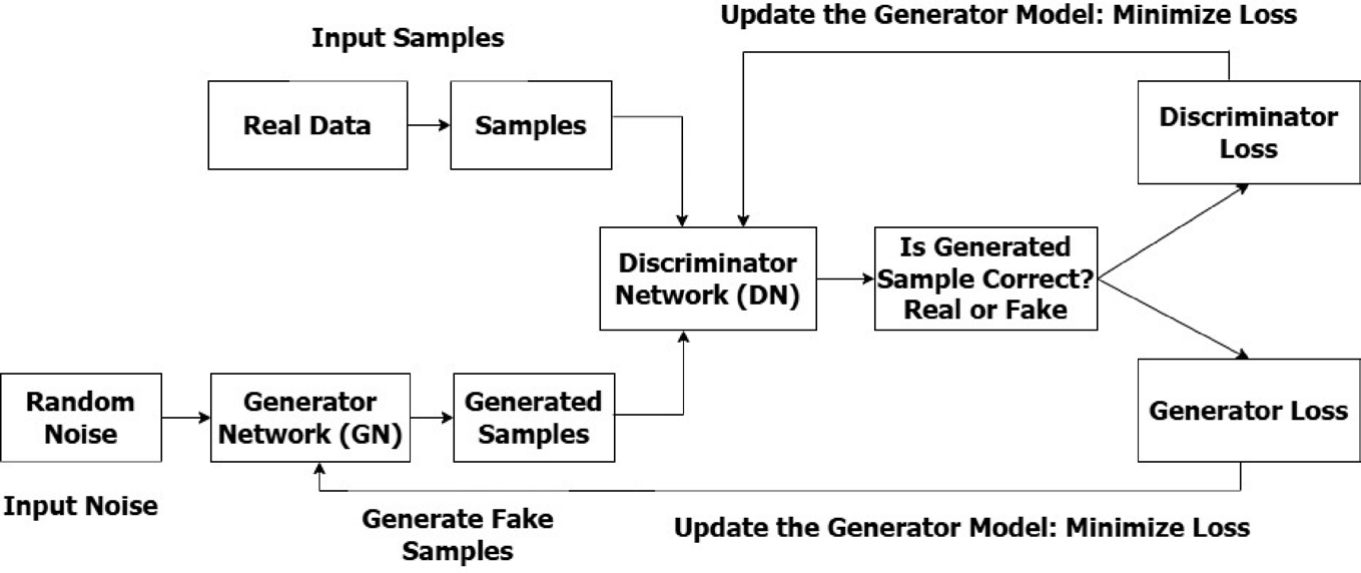
GAN architecture.

Initially proposed by Goodfellow et al.^51^, Generative Adversarial Networks (GANs) are a class of deep learning (DL) models capable of generating novel samples analogous to the training samples. The fundamental concept of GANs is the concurrent training of multiple networks: a generator that produces novel training examples and a discriminator that differentiates between authentic and generated data. The generator network improves its performance by attempting to deceive the discriminator, whereas the discriminator advances by correctly distinguishing between genuine and generated data. This repeated training procedure persists until a balance is achieved between the adversarial loss of the generator and the discriminator. Consequently, a generator that can produce samples analogous to the original, authentic data has been developed. The architecture of the GAN is presented in Fig. 5.

Generative Adversarial Networks (GANs) have been utilised in numerous studies, including Natural language processing (NLP) and computer vision. GANs can learn to produce realistic images by training on a large dataset of authentic images. The generator module can subsequently create fresh pictures that resemble the training samples. In NLP generation, GANs can learn to produce novel text based on training data.

Nevertheless, GANs are seldom used for creating tabular data or CSV files. One rationale for this is that acquiring CSV data requires more structure than natural language or image generation. In structured data, the associations among variables must be preserved, and the data must be generated from the training data. This signifies that the generated data must have statistical characteristics analogous to those of the original dataset, ensuring the preservation of each attribute’s distribution and its interrelations. If the original dataset demonstrates a particular relationship among two or more attributes, the fabricated data should similarly reveal a matching correlation or statistical pattern. Similarly, if the original dataset specifies a range for a particular feature, the generated data must exhibit a similar range. A major task in utilising GANs to generate structured data is the complexity of handling categorical or discrete variables. Categorical variables, unlike continuous attributes, can take a finite set of distinct values, which hinders the accurate modelling of their distribution.

Alongside these technical obstacles, there is the problem of data sparsity, where some feature combinations may be exceedingly rare in the training dataset. In such instances, a GAN may struggle to produce samples that align with the training data, resulting in suboptimal performance.

Generating structured data with GANs is a complex endeavour that necessitates meticulous attention to data representation, model architecture, and training methodologies. Although GANs have demonstrated encouraging results in computer vision and natural language generation, their efficacy in generating tabular data remains an active area of investigation. Furthermore, classic GANs may face training difficulties, including vanishing gradients and mode collapse (Radford A. et al.^52^; Salimans, T. et al.^53^), resulting in suboptimal training performance and a lack of diversity in generated samples. Nonetheless, numerous significant strategies and advancements in this domain have arisen to tackle these issues^54,55^.

**Pseudocode:**
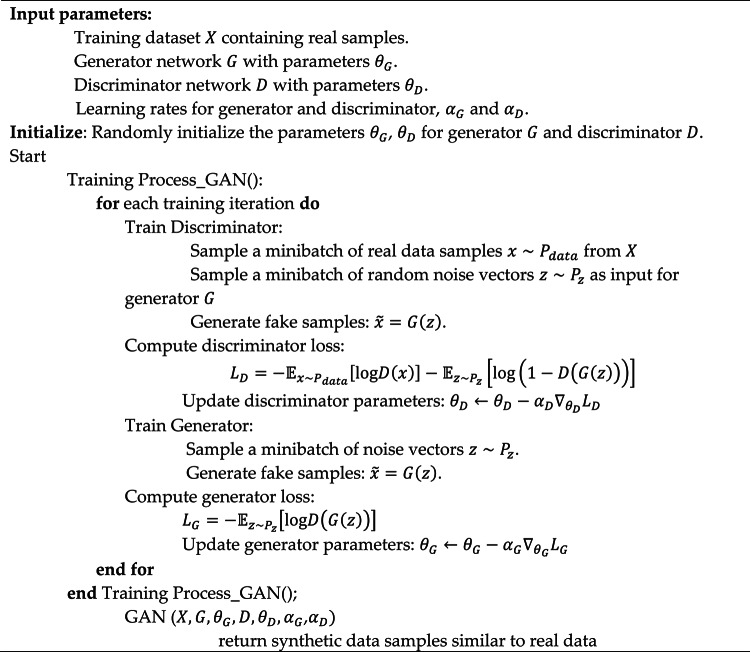
Generative adversarial network (GAN) for synthetic data generation.

### Architecture of generator and discriminator networks

The generator and discriminator networks are fully connected feed-forward neural networks. The generator takes a random noise vector of dimension 16 as input and passes it through two hidden layers, each with 64 neurons and ReLU activation. The output layer is a fully connected layer that maps the learned representations to the dataset’s feature space.

The discriminator network receives input samples of the same dimensionality as the dataset and processes them through two hidden layers, each with 64 neurons and a LeakyReLU activation (α = 0.2). The final output layer consists of a single neuron with a Sigmoid activation function that outputs the probability that the input sample is real or synthetic.

### Hyperparameter values

Hyperparameter optimisation is one noteworthy aspect in any neural network training process. So, hyperparameter optimisation will improve the training process and produce good synthetic data. By choosing appropriate settings for the learning rate, batch size, and architecture-specific parameters, the GAN can provide more realistic and varied outputs. Table 7 presents the hyperparameters modified in our research. We determined an appropriate range of values for each hyperparameter. The specified ranges were derived from recommendations in deep-learning research about melanoma. Both the generator loss curves and discriminator loss curves are presented in Appendix A.

**Table 7:** Generator and discriminator hyperparameters with search ranges and optimal values.

Hyperparameter	Range	Optimal value	
		Generator network	Discriminator network
Epochs	{20,1024}	300	300
Learning rate	{0-1}	0.0002	0.0002
Batch size	{16-512}	32	32
Activation function	{Tanh, Sigmoid, ReLU, Leaky ReLU, Linear}	ReLU	ReLU
Loss function	Binary Cross Entropy:BCE()	Binary Cross Entropy	Binary Cross Entropy
Noise size	{16,1024}	64	64

The GAN model was trained using a noise dimension of 16, a batch size of 32, and 300 epochs. The generator and discriminator each consist of two hidden layers with 64 neurons per layer. The generator network uses the ReLU activation function, while the discriminator uses LeakyReLU with a negative slope coefficient (α) of 0.2, followed by a Sigmoid activation function in the output layer. Both the network generator and the discriminator are optimised with the Adam optimiser at a learning rate of 0.0002 and trained with the Binary Cross-Entropy (BCE) loss function.

#### Evaluating the quality of synthetic data

Evaluating and ranking generative models’ synthetic data is an extension of assessing and evaluating synthetic datasets. For one model to be generally superior to others, it surpass the others in a comparative assessment of quantifiable performance measures (e.g., utility) across multiple datasets, rather than on a single dataset. To evaluate the quality of the generated synthetic data, we have used the metrics, viz. Maximum Mean Discrepancy (MMD) Score, Kernel Density Estimation (KDE), Earth Mover’s Distance (EMD), Correlation mapping and t-SNE^56–58^. The computed values of the above-stated metrics across the three datasets are presented in Appendix B.

#### Proposed experimental workflow

As shown in Fig. 1, the experimental workflow begins with the acquisition of a raw water-quality dataset, which is subsequently partitioned into training and test sets using a stratified 80–20 split. The test set is isolated and remains untouched through the experiments to prevent data leakage and ensure unbiased evaluation. To address the class imbalance, oversampling techniques such as SMOTE, MAHAKIL, and GAN-based methods are applied exclusively to the training set. The resulting balanced training set is used to train the RF, GB, LR and XGB models. To ensure robustness and reduce variance in performance estimates, a tenfold cross-validation strategy is employed on the training data, in which models are trained and validated across folds before final evaluation. Therefore, each model is trained on the balanced data, with a particular focus on improving minority-class prediction. The trained models are tested on the original, unmodified set using multiple metrics, including accuracy, precision, recall, F-score, AUPRC, and MCC. Finally, a comparison is conducted across different oversampling methods and classifiers to assess their effectiveness, resulting in a fair evaluation of both model performance and the utility of synthetic data.

The computational experiments were conducted on a Windows 11 machine with the following configuration: Windows version 25H2, × 64-based architecture. The device is outfitted with an 11th Generation Intel (R) Core (TM) i5-1135G7 processor, including achieving a peak clock speed of 2.4 GHz.

The machine learning and statistical analysis experiments were conducted in Google Colab, an open platform that provides access to robust computational resources. The setup utilised Python version 3.12.13, along with numerous essential libraries to support analysis and model construction. Included was Scikit-learn version 1.6.1 for implementing machine learning methods. Pandas version 2.2.2 and NumPy version 2.0.2 were utilised for data manipulation, whereas Matplotlib version 3.10.0 was applied for visualisation. Statistical tests and analyses were performed utilising SciPy version 1.16.3 and Statsmodels version 0.12.2. These programs were essential for establishing robust machine learning workflows and precise statistical evaluations.

## Results and discussion

Water quality is conventionally assessed using standards derived from laborious laboratory analysis. We examined numerous machine learning methodologies for their estimation and identified multiple studies that employed them. To evaluate the efficacy of various classifiers, a classification report is generated for each. Tables 8, 9, and 10 illustrate a comparative analysis of performance indicators among the trained models. The findings demonstrate that, in the majority of simulated Water Quality classification and prediction tasks, the Random Forest, XGBoost, and GB models excelled, achieving the highest accuracy and reasonable AUC values. The LR model exhibited worse performance than the other models. We have implemented the RFC, LR, GB, and XGB models in Python (version 3.8.9) using Anaconda 3 on an individual-user platform. For the development of the aforementioned classifier models, the NumPy, Pandas, and sci-kit-learn libraries are utilised, and the ROC AUC curves are generated using the Seaborn and Matplotlib libraries.

**Table 8:** Accuracy values of the model across the datasets using SMOTE oversampling.

Dataset/ implemented oversampling method	Model	Accuracy	Precision (minority class)	Recall (minority class)	F1-score (minority class)	AUC -PR	Balanced accuracy	Brier score
Drinking water Final dataset (Baseline)	LR	97.91	0.963	0.98	0.97	0.985	97.96	0.0199
	RF	98.61	0.981	0.98	0.98	0.999	98.51	0.0087
	XGB	99.30	0.981	1	0.99	0.998	99.44	0.0068
	GB	99.30	0.981	1	0.99	0.981	99.44	0.0069
Drinking water Final_Dataset/ SMOTE	LR	98.95	98.94	98.94	98.94	0.9903	98.95	0.009
	RF	98.95	98.94	98.94	98.94	0.9998	98.95	0.005
	XGB	99.47	98.95	100	99.47	0.997	99.48	0.004
	GB	99.47	98.95	100	99.47	0.9895	99.48	0.005
Drinking Water Final_Dataset/ MAHAKIL	LR	91.19	95.40	86.45	90.71	97.73	91.16	0.0631
	RF	97.92	98.93	96.87	97.89	99.78	97.92	0.0203
	XGB	97.92	98.93	96.87	97.89	99.43	97.92	0.0209
	GB	97.92	98.93	96.87	97.89	99.46	97.92	0.0195
Drinking Water Final_Dataset/ GAN	LR	98.96	100	97.72	98.85	99.88	98.86	0.0102
	RF	100	100	100	100	100	100	0.0026
	XGB	99.48	98.87	100	99.43	100	99.52	0.0023
	GB	100	100	100	100	100	100	0.0026

**Table 9:** Accuracy values of the model across the data sets using MAHAKIL oversampling.

Dataset/implemented oversampling method	Model	Accuracy	Precision (minority class)	Recall (minority class)	F1- score (minority class)	AUC -PR	Balanced accuracy	Brier score
Water potability dataset (baseline)	LR	63.81	0.387	0.496	0.435	0.506	50	0.231
	RF	66.41	55.483	36.28	0.438	0.59	59.88	0.214
	XGB	63.35	49.289	43.88	0.435	0.506	59.14	0.231
	GB	67.17	58.730	31.22	0.408	0.594	59.39	0.214
Water potability dataset/ SMOTE	LR	52.19	0.49	0.638	0.556	0.52	52.83	0.224
	RF	71.83	0.690	0.728	0.708	0.77	71.89	0.199
	XGB	65.58	0.62	0.670	0.646	0.71	65.66	0.248
	GB	63.07	0.59	0.659	0.627	0.66	63.23	0.224
Water potability dataset/ MAHAKIL	LR	50.06	0.47	0.640	0.547	0.50	50.84	0.250
	RF	63.57	0.61	0.603	0.609	0.68	63.40	0.223
	XGB	62.20	0.59	0.617	0.605	0.67	62.17	0.247
	GB	59.44	0.56	0.577	0.572	0.65	59.35	0.231
Water potability dataset/ GAN	LR	63.32	0.59	0.688	0.638	0.64	63.32	0.230
	RF	71.58	0.77	0.563	0.651	0.80	71.58	0.179
	XGB	69.21	0.69	0.614	0.652	0.79	69.21	0.202
	GB	71.58	0.77	0.558	0.649	0.79	71.58	0.186

**Table 10:** Accuracy values of the model across the data sets using GAN oversampling.

Dataset/ implemented oversampling method	Model	Accuracy	Precision (minority class)	Recall (minority class)	F1- score (minority class)	AUC- PR	Balanced accuracy	Brier score
Water quality analysis dataset (baseline)	LR	89.75	0.730	0.285	0.410	0.55	63.50	0.079
	RF	95.68	0.964	0.68	0.797	0.91	83.82	0.036
	XGB	97.06	0.922	0.835	0.876	0.94	91.25	0.023
	GB	95.75	0.934	0.71	0.806	0.92	85.14	0.031
Water quality analysis dataset/ SMOTE	LR	98.95	0.98	0.98	0.989	0.99	98.95	0.0090
	RF	98.95	0.98	0.98	0.989	0.99	98.95	0.0053
	XGB	99.47	0.98	1	0.994	0.99	99.48	0.0049
	GB	99.47	0.98	1	0.994	0.98	99.48	0.005
Water quality analysis dataset/ MAHAKIL	LR	69.94	0.724	0.641	0.680	0.73	69.92	0.202
	RF	90.89	0.928	0.885	0.906	0.96	90.89	0.077
	XGB	97.07	0.983	0.957	0.970	0.99	97.06	0.020
	GB	92.87	0.935	0.920	0.927	0.98	92.87	0.054
Water quality analysis dataset/ GAN	LR	92.48	0.916	0.934	0.925	0.90	92.48	0.063
	RF	97.24	0.995	0.949	0.971	0.99	97.24	0.021
	XGB	97.88	0.988	0.968	0.978	0.99	97.88	0.015
	GB	97.28	0.991	0.953	0.972	0.99	97.27	0.020

### ROC-AUC analysis

The ROC curve has been utilised to measure the accuracy of groundwater quality evaluation models. The ROC is a comparative metric that signifies the likelihood of a class using the Boolean approach. The vertical axis of the ROC curve indicates the true positive rate, and the horizontal axis denotes the false positive rate. AUC represents the area under the ROC curve, with values ranging from 0 to 1. The elevated values signify the models’ superior performance. A number approaching 1.0 indicates a high accuracy of the anticipated model. The ROC curve approach utilises the AUC as a key metric for evaluating model performance, with values ranging from 0.5 to 1.

The intrinsic validity of a diagnostic test can be evaluated using the AUC, sensitivity, and specificity statistics. An AUC of 1 indicates that the test can differentiate between non-potable and non-potable ground cases with complete accuracy. The non-appearance of false positives or negatives indicates equal sensitivity and specificity. This is quite improbable. As test performance improves, the AUC approaches one. An AUC value of 0 indicates that the test erroneously classified all potable water as not potable and all not potable water as potable; hence, the minimum acceptable AUC value should be 0.5. When the test findings are inverted, the area that was formerly 0 becomes 1, rendering an otherwise entirely defective test accurate. The model’s performance in this context must exceed an AUC of 70%.

In the current study, we assessed the classifiers’ predictive performance using the AUC. The prediction was then compared with the actual water quality classes to determine the correct classification. According to Walter^59^, the AUC ranges from 0 (no discriminatory power) to 1 (excellent discrimination). The performance of the classifier could be assessed using five key interpretations. Hosmer and Lemeshow^60^ state that the following interpretations are possible:

- Outstanding discrimination (AUC ≥ 0.9): all classes can be correctly classified by the classifier model.
- Excellent discriminating capability: the classifier has a high probability of accurately classifying data (AUC = 0.8 to 0.9).
- Satisfactory discrimination: classifier performance is satisfactory, with a small number of incorrect classifications (AUC = 0.70 − 0.79).
- The model lacks discrimination capabilities (AUC ≤ 0.5), meaning the classifier cannot effectively differentiate between classes.
- Poor discrimination (AUC = 0.5 − 0.7): the classifier is classifying features with increased incorrect classification.

### Sensitivity analysis of models over the oversampled datasets-SMOTE

**Fig. 6:**
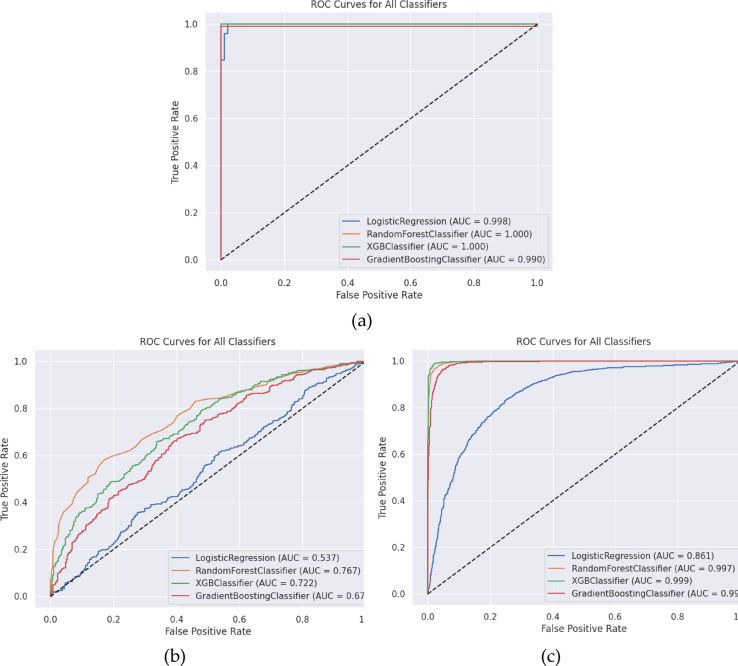
ROC curve analysis of models over the oversampled datasets using SMOTE, (**a**) Drinking Water_Final_Dataset using SMOTE (**b**) Water potability dataset using SMOTE, (**c**) Water quality analysis dataset using SMOTE.

We analysed four ML models, viz. LR, RF, XGBoost, and GB Classifier to classify water quality using the oversampled Drinking Water Final Dataset with SMOTE (Synthetic Minority Oversampling Technique). The models were evaluated based on their Receiver Operating Characteristic (ROC) curves and Area Under the Curve (AUC) scores, which measure their ability to discriminate between potable and non-potable classes. RF and XGB achieved perfect performance with an AUC score of 1.0, indicating flawless classification capability. Followed by GB Classifier with an AUC = 0.9, LR with AUC = 0.9, Sub-Fig. 6a.

Using the oversampled Water Potability dataset with SMOTE (Synthetic Minority Oversampling Technique), we evaluated the effectiveness of ML models in predicting water quality. From Sub-Fig. 6b, it is observed that the Random Forest achieves reasonable performance with an AUC of 0.7, followed by XGBoost with an AUC of 0.7. Next come the GB Classifier (AUC 0.6) and the LR model (AUC 0.5). This analysis indicates that the ML model’s accuracy is considerably higher on the oversampled dataset than on the non-oversampled Water Potability (Baseline) dataset.

Using the oversampled Water Quality Analysis dataset with SMOTE (Synthetic Minority Oversampling Technique), the performance of the four models is evaluated via ROC curves. From Sub-Fig. 6c, it is observed that the XGB model outperformed the remaining models, with an AUC value of 0.999%, followed by the RF model (AUC = 0.997), XGB (AUC = 0.992), and LR (AUC = 0.861). From this analysis, it is noticed that all the models achieved good AUC values for water quality classification.

### Sensitivity analysis of models over the oversampled datasets-MAHAKIL

**Fig. 7:**
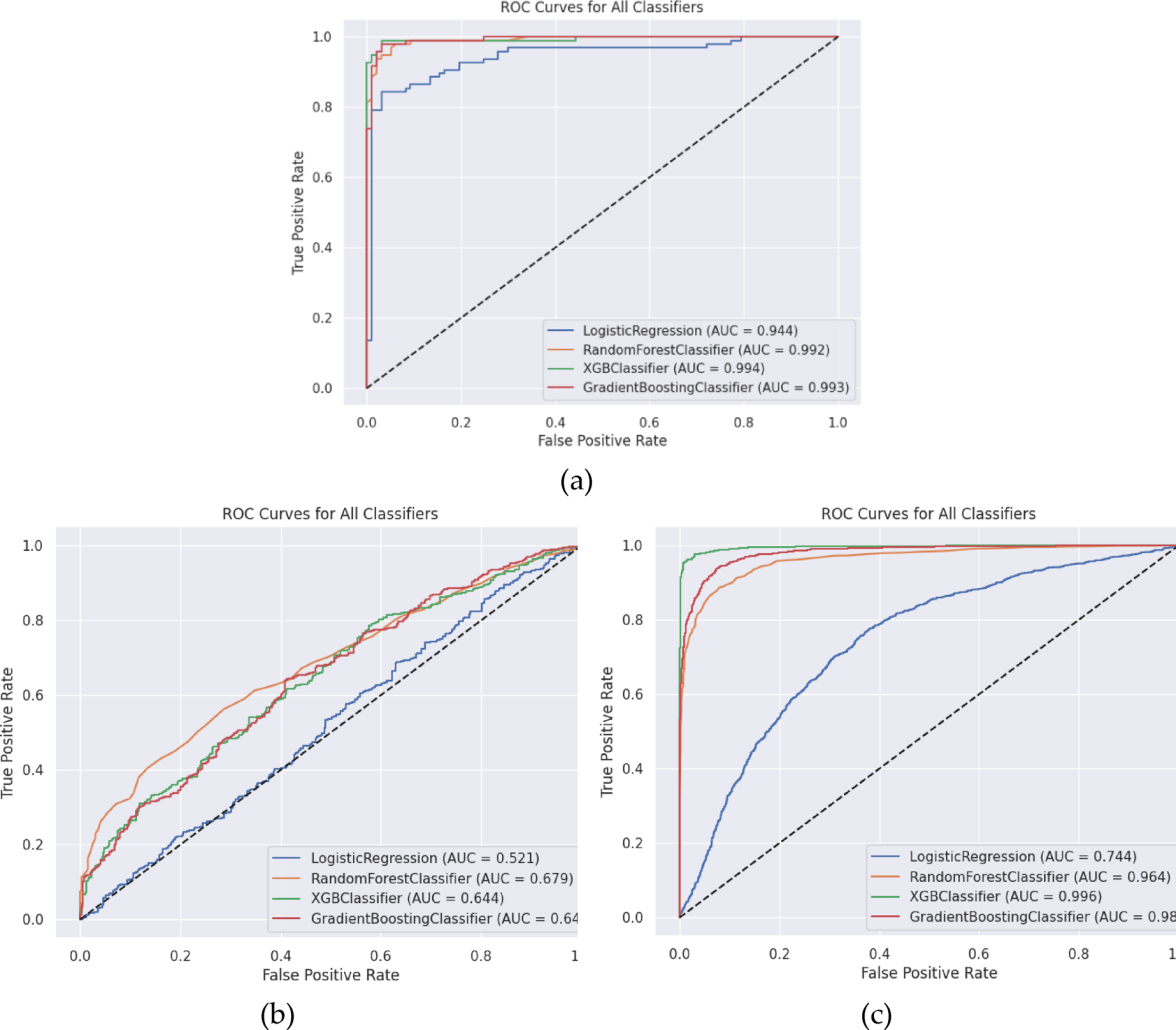
ROC curve analysis of models over the oversampled datasets using MAHAKIL, (**a**) Drinking water final dataset using MAHAKIL (**b**) Water potability dataset using MAHAKIL, (**c**) Water quality analysis dataset using MAHAKIL.

We analysed four machine learning models—LR, Random Forest, XGBoost (Extreme Gradient Boosting), and GBM Boosting Classifier—to classify water quality using the oversampled Drinking Water Final Dataset with MAHAKIL. The models were evaluated based on their Receiver Operating Characteristic (ROC) curves and Area Under the Curve (AUC) scores, which measure their ability to discriminate between classes. Random Forest achieved better performance with an AUC score of 0.992, indicating nearly flawless classification capability. XGB with an AUC value of 0.994, LR with an AUC of 0.994, and GB with an AUC of 0.993. Sub-Fig. 7a. This analysis indicates that all models perform well, with strong classification capability.

Using the oversampled Water Potability dataset with MAHAKIL, we evaluated the effectiveness of ML models in forecasting water quality. Random Forest achieved AUC values of 0.679. Next come the Gradient Boosting Classifier (AUC 0.646), the XGB Classifier (AUC 0.644), and the LR model (AUC 0.521) (Sub-Fig. 7b). This analysis indicates that the RF model outperforms the LR model. All four models treat the potable classes as not potable and vice versa.

Using the oversampled water-quality analysis dataset with MAHAKIL, the performance of the four models is evaluated via ROC curves. From Sub-Fig. 7c, it is observed that the XGB model outperforms the remaining models, with an AUC value of 0.996%, followed by the GB model (AUC = 0.981), RF (AUC = 0.964), and LR (AUC = 0.774).

### Sensitivity analysis of models over the oversampled datasets-GAN

**Fig. 8:**
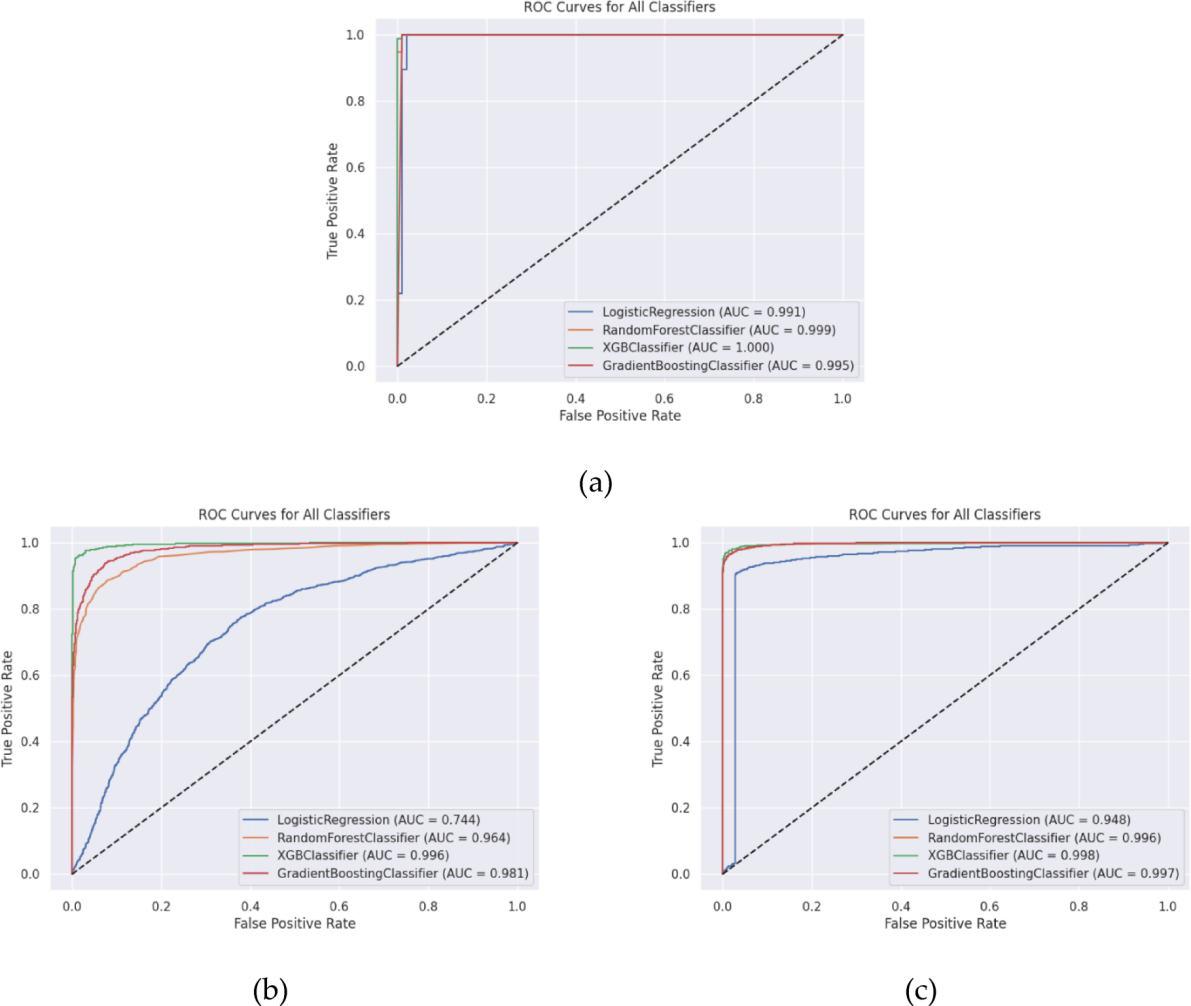
ROC curve analysis of models over the oversampled datasets using GAN, (**a**) Drinking Water_Final_Dataset using GAN (**b**) Water potability dataset using GAN, (**c**) Water quality analysis dataset using GAN.

We have analysed four ML models-LR, RF, XGB, and GB Classifier to classify water quality using the oversampled Drinking Water Final Dataset with GAN (Generative Adversarial Network for Synthetic Data Generation). As presented in Sub-Fig. 8a, the three models—RF, XGB, and GB—attain AUC values greater than 0.95, closely followed by the LR model with an AUC of 0.991. These AUC values indicate that all four models are the best-fit models, with a good level of agreement between the predicted and original class labels.

Using the oversampled Waterpotability dataset with GAN, we evaluated the effectiveness of machine learning models in predicting water quality. Both XGB and GB achieved AUCs of 0.996 and 0.981, respectively. Next, the RF Classifier achieves an AUC of 0.964, while the LR model attains an AUC value of 0.774 in Sub-Fig. 8b. These AUC values indicate that the RF, XGB, and GB models achieve good accuracy.

Using the oversampled water-quality analysis dataset with a GAN, the performance of the four ML models is assessed via the ROC curve. From Sub-Fig. 8c, it is observed that the XGB model outperforms the other models, with an AUC of 0.998, followed by the GB model (AUC = 0.997), RF (AUC = 0.996), and LR (AUC = 0.948). These AUC values, ranging from 0.948 to 0.999, indicate that the four models have strong predictive power for groundwater classification on the oversampled water-quality analysis dataset using GAN.

### Ablation study

An ablation study is a crucial tool for assessing the influence of particular components or techniques within a machine learning model. In this study, the impact of the three oversampling techniques is assessed by implementing the 4 classifiers over the synthetic datasets generated. The ablation study includes the following.

1. Performance Evaluation of the four models on the Oversampled (SMOTE) Datasets Vs. Original Dataset (Baseline).
2. Performance Evaluation of the four models on the Oversampled (MAHAKIL) Datasets Vs. Original Dataset (Baseline).
3. Performance Evaluation of the four models on the Oversampled (GAN) Datasets Vs. Original Dataset (Baseline).

#### Performance evaluation of models on the drinking water final oversampled dataset using SMOTE/MAHAKIL and GAN

We used a tenfold cross-validation strategy and an independent test set assessment to make sure that the model could be generalised^61^. We also used several classifier performance metrics, such as Balanced Accuracy, ROC-AUC, Accuracy, F1-Score, Precision, Recall, Average Accuracy, and Brier scores. The computed values of the machine algorithms (testing phase) for the synthetic Drinking Water Final datasets generated by SMOTE, MAHAKIL, and GAN (Drinking Water Final Dataset/SMOTE, Drinking Water Final Dataset/MAHAKIL, and Drinking Water Final Dataset/GAN) are presented in Table 8.

The considered model’s performance on the oversampled Drinking Water Final Dataset with SMOTE indicates that the XGB and GB models outperformed the other models (XGB: accuracy = 99.47, Precision = 0.9, Recall = 1.0, F1 score = 99.5, Balanced Accuracy = 99.48, and Brier score = 0.004).GB: accuracy = 99.47, Precision = 0.98, Recall = 1.0 and F1 score = 99.4, Balanced Accuracy = 99.48 and Brier score = 0.005) followed by the Random Forest model (attains 98.95% accuracy, Precision = 0.989, Recall = 0.989 and F1 score = 0.989 and Brier score = 0.005), followed by LR (accuracy = 98.95, Precision = 0.9, Recall = 0.9 and F1 score = 0.9 and Brier score = 0.009). Previous research by Patel et al.^49^ also showed that the use of data augmentation and ensemble models provides better groundwater classification and prediction. A similar kind of study by K. K., Krishnan S., and Manikandan R.^62^ utilised SMOTE to balance the dataset. Yee Wong W et al.^63^ suggest a modified RF by integrating the SMOTE into the traditional Random Forest methodology.

The performance of the models on the oversampled Drinking Water Final Dataset with MAHAKIL showed that the XGB, RF, and GB models achieved 97.92% accuracy. The XGB and RF models recorded Brier scores of 0.0209 and 0.0203, respectively, and the GB model’s Brier score (0.019) is slightly lower than those of the XGB and RF models. The LR model attains a reasonable accuracy of 91.19%, with Precision = 0.90, Recall = 0.8, F1 score = 0.9, Balanced Accuracy = 91.16, and Brier score = 0.0631. The performance of the models on the oversampled Drinking Water Final Dataset with GAN highlights that the RF and GB models achieved the highest accuracy of 100%, with corresponding Brier scores of 0.0026. The XGB model attained99.48% accuracy, and its balanced accuracy is 99.53%. The LR model also achieves a high classification accuracy of 98.96%.

The above comparative analysis aligns with the visual inspection of the synthetic data generated using SMOTE as presented in Appendix B. The Drinking Water Final dataset, oversampled using SMOTE and GAN, will improve the classifier’s prediction accuracy.

The model’s performance on the Drinking Water Quality Final Dataset (Baseline-without oversampling) achieved near-perfect accuracy (XGB and GB accuracies of 99.30%) and an AUCPR ≈99%, indicating that the dataset itself is highly separable. Further, it allows models to achieve high predictive performance.

Models like XGB and GB attain perfect accuracy for specific splits when SMOTE and GAN-based oversampling approaches were applied. This improvement is attributed to balanced representation of the minority class, which reduces the class imbalance and allows the model to learn class boundaries.

To ensure the validity of the results, oversampling was performed on the training set only to prevent data leakage. The test set remained untouched. Moreover, stratified splitting and various random seeds were employed to validate the consistency and robustness of the findings. Therefore, the observed near-perfect and perfect performance is not an artefact of overfitting but rather a reflection of the dataset characteristics combined with effective class balancing.

#### Performance evaluation models on the water potability oversampled dataset using SMOTE/MAHAKIL and GAN

Table 9 presents a comparative analysis of performance indicators across the trained models on the Water Potability dataset oversampled with SMOTE, MAHAKIL, and GAN (Water Potability Dataset/SMOTE, Water Potability Dataset/MAHAKIL, and Water Potability Dataset/GAN). From Table 8, the RF model attained an accuracy of 71.8%, with Precision = 0.69, Recall = 0.7, F1 score = 0.7, Balanced Accuracy = 71.90, and Brier score = 0.199. Followed by the XGB model with 65.6% accuracy (Precision = 0.62, Recall = 0.7 and F1 score = 0.7, Balanced Accuracy = 71.89 and Brier score = 0.248), GB with 63.07% accuracy ( Precision = 0.59, Recall = 0.659 and F1 score = 0.627, Balanced Accuracy = 63.23 and Brier score = 0.224) and the LR model performed poorly with an accuracy of 52.19% accuracy (Precision = 0.49, Recall = 0.64 and F1 score = 0.638, Balanced Accuracy = 52.83 and Brier score = 0.224).

Similarly, the model’s performance on the Water potability dataset, oversampled with MAHAKIL, showed that the RF model’s accuracy was 63.57% (Precision = 0.61, Recall = 0.60, F1 score = 0.60, Balanced Accuracy = 63.40, and Brier score = 0.223). Followed by the XGB with 62.2% accuracy (Precision = 0.59, Recall = 0.61 and F1 score = 0.605, Balanced Accuracy = 62.17 and Brier score = 0.247). Surprisingly, the GB and LR models performed poorly, achieving accuracies of 59.4% and 50.06%, respectively. The LR and GB models’ performance is low, and this is critical, as hazardous water that is erroneously classified could be potable by humans. Numerous studies have examined the superiority of XGBoost and ensemble models in groundwater classification and prediction, revealing variability in model performance across different investigations (Samir Patel et al.^64^; Ansari AT et al.^65^).

On the Water potability dataset oversampled with a GAN, the RF and GB models achieved a reasonable accuracy of 71.58% (Precision = 0.7, Recall = 0.5, F1 score = 0.6, Balanced Accuracy = 71.58, and Brier score = 0.248); the RF Brier score = 0.179 and the GB Brier score = 0.186. Even though the RF and GB models achieved the same accuracy, the RF model’s Brier score is slightly better than the GB model’s. The XGB model attained 69.21% accuracy (Precision = 0.6, Recall = 0.6, F1 score = 0.6, Balanced Accuracy = 69.21, and Brier score = 0.202). Compared with the RF, GB, and XGB models, the LR model underperforms, achieving an accuracy of 63.32%.

The above comparative analysis aligns with the visual inspection of the synthetic data generated using SMOTE as presented in Appendix B. The Water Portability dataset, oversampled using SMOTE, MAHAKIL and GAN, is not suitable for ML model training.

#### Performance evaluation models on the water quality analysis oversampled dataset using SMOTE/MAHAKIL and GAN

Table 10 illustrates a comparative analysis of performance indicators among the trained models on the oversampled dataset for Water Quality Analysis using SMOTE, MAHAKIL, and GAN (Water Quality Analysis/SMOTE, Water Quality Analysis/MAHAKIL, and Water Quality Analysis/GAN).

From the model comparison analysis over the oversampled Water Quality Analysis using the SMOTE, it is noted that XG and GB exhibit outstanding accuracy in predicting water quality classification (accuracy = 99.47, F1-score = 0.99, recall = 1.0, and precision = 0.98, Balanced accuracy = 99.48), and Brier scores are 0.0049 and 0.005. Followed by the RF and LR with 98.95% accuracy (F1-score = 0.98, recall = 0.98, and precision = 0.98, Balanced accuracy = 98.95, and Brier scores are 0.0053 and 0.0090, respectively.

In the oversampled Water Quality Analysis using the MAHAKIL, the XGB model showed superior performance, achieving 97.07% accuracy, with F1-score = 0.9, recall = 0.9, precision = 0.92, balanced accuracy = 97.06, and Brier score = 0.020. Followed by the GB model with 92.87% accuracy. Next comes the RF model with 90.89% accuracy. Compared with the XGB, GB, and RF models, the LR model achieved a lower accuracy of 69.94%. Compared with other traditional methods, the experimental findings demonstrate that the boosting and bagging model may increase the accuracy of classifying water quality classes and capture the intricate non-linear relationship between the input and output data. Numerous studies have corroborated this; in a tropical setting, XGB models predict the water quality index with good performance accuracy when compared to other traditional approaches^39,66–73^. Furthermore, because the RF model is easy to understand and apply, it works well for determining the WQI class label. Data about water quality can be facilitated, analysed, and categorised by it^74^.

In the oversampled Water Quality Analysis using the GAN, the XGB model emerged as the superior model, achieving 97.88% accuracy (F1-score = 0.97, recall = 0.96, precision = 0.98, Balanced accuracy = 97.88, and Brier score = 0.015). Next come the GB and RG models, with 97.2% accuracy; the Balanced accuracies are 97.24% and 97.27%, respectively. The Brier scores for the GB and RF modes are 0.020 and 0.021, respectively. Lastly, the LR model attained 92.48% accuracy, 92.48% Balanced accuracy, and a Brier Score of 0.063. To further validate the model’s accuracy, we run the 4 ML models using 5 random seeds across the oversampled datasets. For each classifier and the oversampling method, we report the mean and standard deviation across the 5 runs and the tabulated values are presented in Appendix C.

This study highlights the following observations:

- The synthetic data generation methods are used to balance the considered 3 datasets.
- The visual inspection (KDE plot, Correlation map and t-SNE map) reveals valuable insights- The SMOTE and GAN-generated synthetic data are reasonable across the 3 datasets. The MAHAKIL oversampled synthetic dataset is not suitable for training ML models.
- The LR, RF, GB and XGB models’ accuracies are high if the sample points preset in the dataset are perfectly linearly separable, even though the data is unbalanced. This observation is true for the Drinking Water Final dataset.

**Fig. 9:**
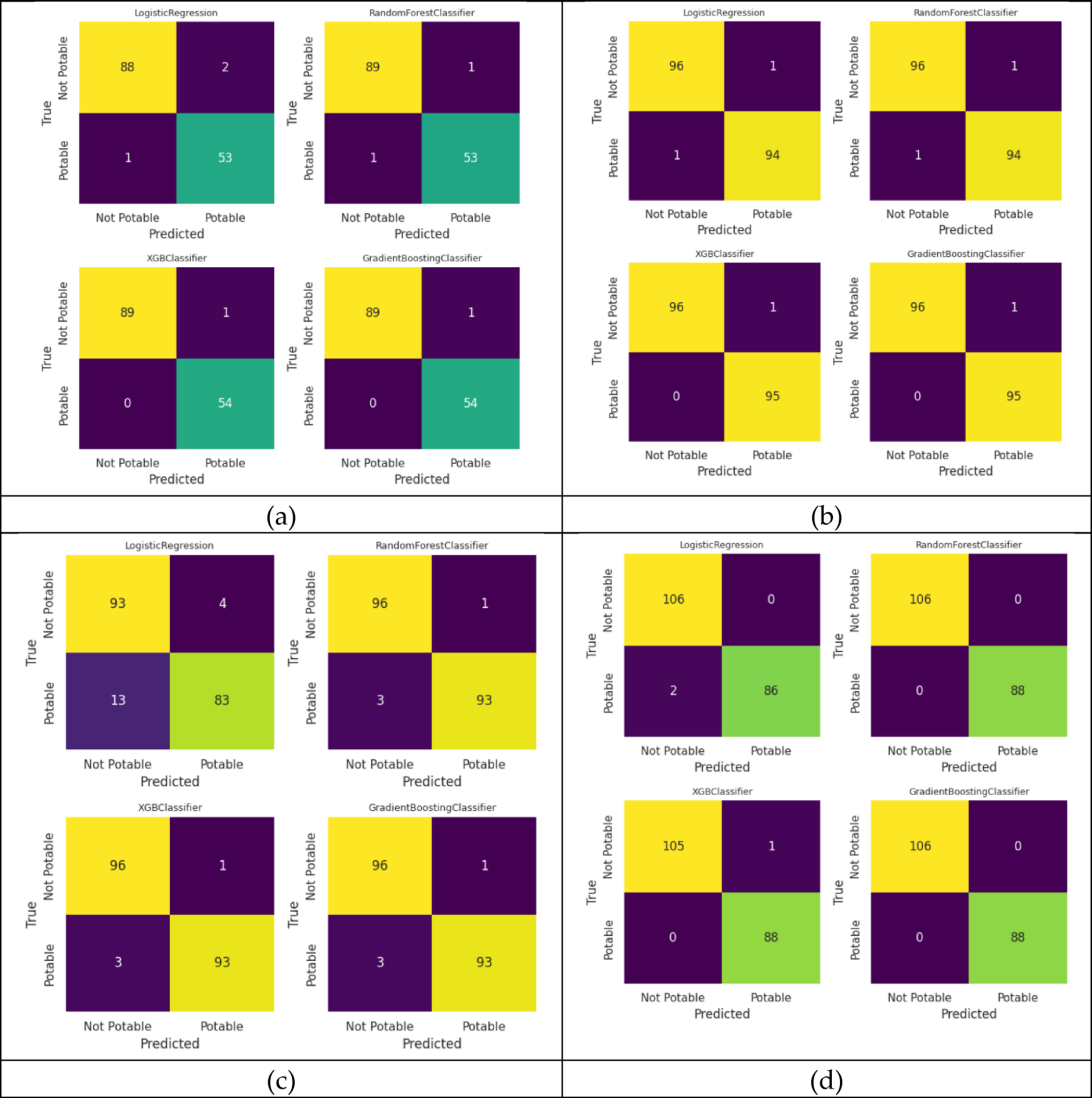
Confusion matrix of applied models on drinking water final dataset, (**a**) Drinking water final without modification, (**b**) Drinking water final oversampled-SMOTE, (**c**) Drinking water final oversampled-MAHAKIL, (**d**) Drinking water final oversampled-GAN.

**Fig. 10:**
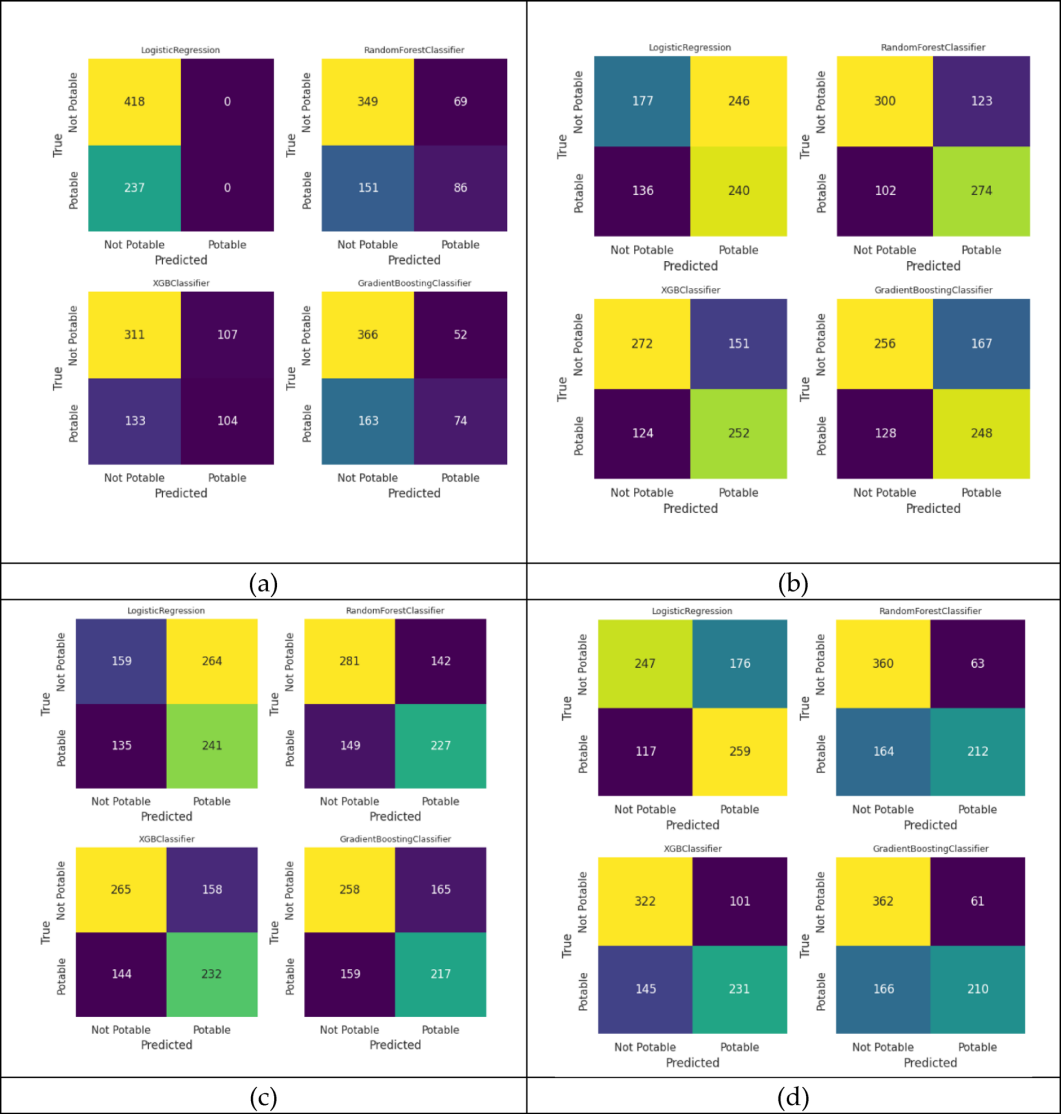
Confusion matrix of applied models on water potability dataset, (**a**) Water potability dataset without modification, (**b**) Water potability dataset-SMOTE, (**c**) Water potability dataset-MAHAKIL, (**d**) Water potability dataset-MAHAKIL.

**Fig. 11:**
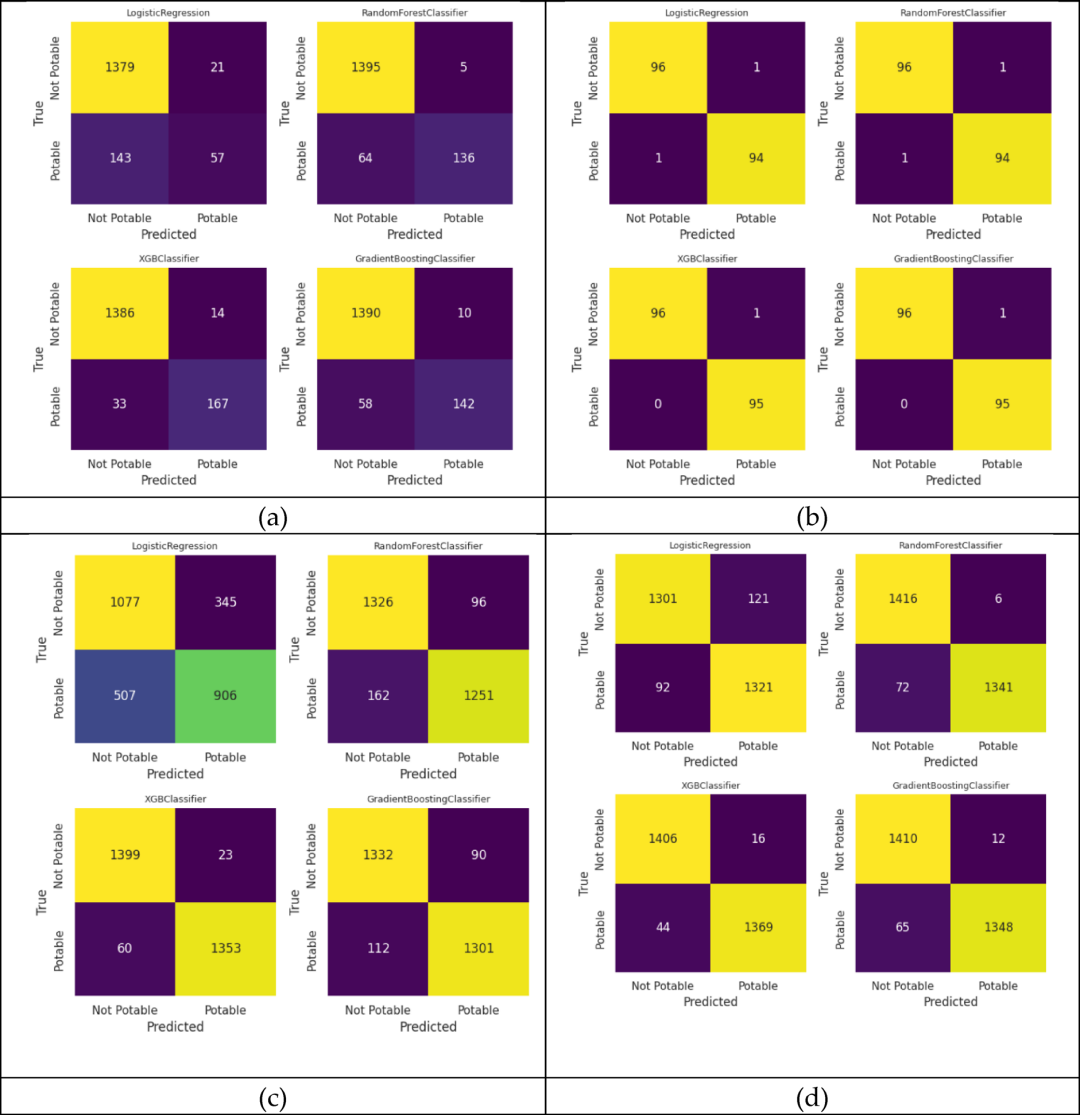
Confusion matrix of applied models on water quality analysis dataset, (**a**) Water quality analysis dataset without modification, (**b**) Water quality analysis dataset oversampled-SMOTE, (**c**) Water quality analysis oversampled-MAHAKIL, (**d**) Water quality analysis dataset oversampled-GAN.

Finally, the performance of four ML models for predicting water quality (potable or non-potable), along with data oversampling techniques, is evaluated using the confusion matrix. Figures 9, 10 and 11 depict the performance of the models on the data sets oversampled using SMOTE, MAHAKIL, and GAN, respectively. The confusion matrix for the water quality classification task is depicted in the same order as presented in Tables 7, 8 and 9, from LR to Gradient Boosting.

From Fig. 9a–d, it is observed that the RF model performed extremely well over the oversampled Drinking water final Dataset using SMOTE. Figure 10a–d shows that the RF, XGB, and GB models performed well (accuracy = 71.58) on the Drinking water final Dataset using the MAHAKIL oversampling approach. From Fig. 11a–d, it is observed that the XGB model achieves an accuracy of 97.28% on the Drinking water final Dataset using the GAN for data balancing. In summary, SMOTE and GAN are identified as effective data balancing methods for achieving accurate performance with the RF, GB, and XGB models across the 3 datasets considered in this study.

#### Comparative study

Table 11 presents a comparative study of SMOTE oversampling techniques with downstream classifiers. For instance, in study^75^, Mounir et al. investigated water quality classification under severe class imbalance by comparing 8 data augmentation techniques across 5 machine learning classifiers. The results of the study demonstrate that the hybrid SMOTE-ENN approach greatly improves the unsafe water detection, achieving a recall of 97.77% and reducing missed contamination cases by over 87%, highlighting its suitability for safety–critical monitoring systems. Similarly, in the study^76^, Shafi et al. employed SMOTE to balance the dataset and thereafter applied machine learning–based classification models for automated water quality assessment, reporting accuracies as high as 98.87%. The results show that data-driven techniques have significant potential.

**Table 11:** Comparative study of the existing approaches.

Refs. & Year	Dataset/source	Oversampling technique	Best predictive model	Accuracy
2025 ^75^	Water quality dataset/Kaggle	SMOTE, ADASYN, Borderline-SMOTE, SMOTE-ENN, MixUp, CTGAN and TVAE	SMOTE-ENN	97.77% (*recall)
2025 ^76^	Raw data from various Indian regions/CPCB, 2025	SMOTE	XGB	98.87%
2025 ^77^	Water quality/Kaggle	SMOTE	XGB	98.92%
2024 ^78^	Water quality dataset/Kaggle	SMOTE	LightGBM	95.06%

Toleva et al.^77^ employed classical machine learning models combined with a customised SMOTE-based sampling strategy to address class imbalance in drinking water quality classification^76^. The XGBoost model achieved an accuracy of 98.92% with significantly improved sensitivity and F1-score. However, the approach relies on a single train–test split for evaluation and is limited to a single public dataset. In another study^78^, Karthick K et al. employed Random Forest, XGBoost, and Support Vector Machine models with SMOTE-based class balancing for WQI prediction using the Kaggle water quality dataset, and LightGBM attain 95.06% accuracy. However, the evaluation is largely based on a limited set of validation settings, without repeated or stratified cross-validation. When SMOTE was applied to the classifiers, performance decreased slightly.

Our findings are in line with the conclusions of Karthick K et al. It can be concluded that not all oversampling techniques will improve the accuracy of underlying classifiers. Among the oversampling techniques, SOMTE outperformed the MAHAKIL and GAN. The downstream classifiers GB and XGB performed well, consistent with^78^.

Models trained on data from a specific region often exhibit poor generalisation when applied to other regions due to climatic conditions, variation in pollution sources, and geographical features. This issue is one major limitation of the comparative investigation^79^.

## Conclusion and future work

This research explored machine learning models and synthetic data generation methods to assess water quality, using different input parameters to address the deficiency of water quality data for classification and prediction. The synthetic data generation methods produce samples similar to the original data values. Comprehensive comparison of the machine learning models over the dataset without modification and the oversampled datasets. The comparative study reveals that the XGB and RF models performed well across the three datasets. The results of the research indicate that data augmentation approaches (synthetic data generation) are a viable solution for the deficiency of water quality data. In the future, we plan to utilise feature engineering alongside optimisation techniques to identify key characteristics that enhance machine learning model performance.

## Supplementary Information

Below is the link to the electronic supplementary material.

Supplementary Material 1

## Data Availability

The datasets generated during and/or analysed during the current study are available from the corresponding author on reasonable request.
