# Supplementary material for: Data driven water quality assessment using machine learning and synthetic data generation
Source: Sci Rep. 2026 Jun 5;16:25661. doi: 10.1038/s41598-026-54560-7 (PMC13478298; doi:10.1038/s41598-026-54560-7)
Supplement: Supplementary file 1 [file 41598_2026_54560_MOESM1_ESM.docx]

**APPENDIX A**

The following Figures A1-A3 depicts the loss curves of the GAN for each oversampled dataset and the Generator and Discriminator loss over Drinking Water Final Dataset are presented in Table T1.

Fig A1. GAN Generator and Discriminator Loss Curves over Drinking Water Final Dataset

Fig A2. GAN Generator and Discriminator Loss Curves over Water potability Dataset

Fig A3. GAN Generator and Discriminator Loss Curves over Water Quality Analysis Dataset

Table T1. Generator and Discriminator loss over Drinking Water Final Dataset

| **Epoch** | **Generator loss** | **Discriminator loss** |
| --- | --- | --- |
| 50 | 0.7112 | 0.7669 |
| 100 | 0.7193 | 0.7290 |
| 150 | 0.7157 | 0.7548 |
| 200 | 0.6323 | 0.9366 |
| 250 | 0.8694 | 0.7424 |
| 300 | 0.9529 | 0.7430 |

**APPENDIX B**

**Synthetic Data Quality Metrics**

The overall utility of synthetic data is evaluated by multiple metrics, such as Maximum Mean Discrepancy (MMD) Score, Kernel Density Estimation (KDE), Earth Mover’s Distance (EMD), Correlation mapping and t-SNE. In total we used 5 metrics to estimate the quality of the synthetic data. We compared these metrics against with the SMOTE, MAHAKIL and GAN oversampling method implemented on Drinking Water Final Dataset, Water Potability Dataset and Water Quality Analysis Dataset.

1. **Maximum Mean Discrepancy (MMD)**

In this study first we evaluated the quality of the synthetic data generated using all three oversampling methods on the Drinking Water Final Dataset, Water Potability Dataset and using Water Quality Analysis Dataset using MMD metric. The MMD value quantifies the distributional distance between the original dataset and synthetic dataset. The MMD score near to 0 always indicate the generated synthetic data is good and vice versa.

From the following Table B1, MMD score (0.006771) of SMOTE method over the Drinking Water Final Dataset is very low. Similarly, the SMOTE oversampling method generates good synthetic data over the Water Potability Dataset with 0.002117 MMD score and in case of the Water Quality Analysis Dataset also the SMOTE oversampling method records a low MMD score (0.000648).

Table B1. Maximum Mean Discrepancy (MMD) scores of the oversampling techniques

| Dataset | OVERSAMPLING METHOD | MMD SCORE |
| --- | --- | --- |
| Drinking Water Final Dataset | **SMOTE** | 0.006771 |
|  | MAHAKIL | 0.081679 |
|  | GAN | 0.504748 |
| Drinking Water Final Dataset | **SMOTE** | 0.002117 |
|  | MAHAKIL | 0.045998 |
|  | GAN | 0.012075 |
| Water Quality Analysis Dataset | **SMOTE** | 0.000648 |
|  | MAHAKIL | 0.018767 |
|  | GAN | 0.0016 |

The Drinking Water Final Dataset oversampled with SMOTE achieves an MMD score of 0.006771, which clearly indicates the very strong similarity range. This indicate that synthetic data generated by SMOTE effectively preserve the underlying data distribution. MAHAKIL records an MMD score of 0.081679, suggesting moderate divergence meaning that some distributional distortion is introduced. GAN based oversampling exhibits significantly higher MMD value of 0.504748, indicating the significant distributional mismatch which may lead to biased or less reliable model training.

Over the Drinking Water Final Dataset, SMOTE produces an MMD score of 0.002117 and which is extremely close to 0 and reflects a near perfect distributional alignment. GAN based oversampling method achieves 0.045998, which still indicates good similarity, though slightly less accurate than SMOTE. MHAKIL with 0.045998, fall in the moderate similarity range, suggesting acceptable but less precise distribution matching.

Over the Water Quality Analysis Dataset oversampled dataset, SMOTE (**0.000648**) and GAN (**0.0016**) both yields near-zero MMD values, demonstrating almost identical distribution between synthetic data and original data. While MAHAKIL records 0.018767, which indicates moderate but acceptable deviation.

2. **Kernel Density Estimation (KDE)**

The Kernel Density Estimation (KDE) is the method of estimating an unknown probability density function by applying a kernel function to each data point and aggregating the divergent regions of the two distributions. Visualizing the KDE distribution is beneficial for acquiring insights into the produced data.

Table B2.KS Statistic and EMD scores of the oversampling techniques over Drinking Water Final Dataset

|  | SMOTE | | | MAHAKIL | | | GAN | | |
| --- | --- | --- | --- | --- | --- | --- | --- | --- | --- |
| Feature | **KS Statistic** | **KS**  **p-value** | **EMD** | **KS**  **Statistic** | **KS**  **p-value** | **EMD** | **KS Statistic** | **KS**  **p-value** | **EMD** |
| pH | 0.186 | 0.000 | 0.195 | 0.117737 | 0.028375 | 0.100411 | 1.0000 | 0.0000 | 7.4403 |
| Sodium | 0.573 | 0.000 | 104.716 | 0.320997 | 0.000000 | 36.31363 | 1.0000 | 0.0000 | 83.5935 |
| Magnesium | 0.500 | 0.000 | 62.444 | 0.298085 | 0.000000 | 22.30603 | 1.0000 | 0.0000 | 55.6137 |
| Calcium | 0.498 | 0.000 | 91.274 | 0.308471 | 0.000000 | 28.95503 | 0.8785 | 0.0000 | 67.0408 |
| Chloride | 0.395 | 0.000 | 158.080 | 0.238602 | 0.000000 | 53.73085 | 0.9560 | 0.0000 | 117.726 |
| Potassium | 0.461 | 0.000 | 13.217 | 0.237562 | 0.000000 | 5.139982 | 1.0000 | 0.0000 | 12.8642 |
| Carbonate | 0.199 | 0.000 | 27.208 | 0.134690 | 0.007711 | 11.66461 | 0.8715 | 0.0000 | 117.660 |
| Sulphate | 0.214 | 0.000 | 36.113 | 0.112535 | 0.040829 | 9.704781 | 1.0000 | 0.0000 | 51.5898 |
| TDS | 0.430 | 0.000 | 427.753 | 0.415272 | 0.000000 | 176.7562 | 0.5070 | 0.0000 | 472.057 |
| EC | 0.400 | 0.000 | 432.142 | 0.267910 | 0.000000 | 199.4253 | 0.9982 | 0.0000 | 977.764 |
| TH | 0.499 | 0.000 | 128.435 | 0.246881 | 0.000000 | 39.42452 | 0.8838 | 0.0000 | 196.241 |
| WQI | 0.676 | 0.000 | 19.755 | 0.298816 | 0.000000 | 7.129753 | 1.0000 | 0.0000 | 54.1873 |
|  | | | | | | | | | |

FigureF1: KDE Plot of Drinking Water Final Dataset oversampled using SMOTE.

**Evaluation of Synthetic Drinking Water Final Dataset:** From Table B2 it is noticed that, for all features, the KS p-values for SMOTE and GAN are consistently 0.00, means that the null hypothesis of identical distributions is rejected. Indicates that both the oversampling methods introduce statistically significant deviations from the original data distribution. MAHAKIL shows slightly higher KS p-values for few parameters (e.g. pH, Sulphate), indicating that distributional differences persist.

In addition to the above, the EMD results also enforce the same, GAN records the maximum EMD values for all features (EC=977.764, TDS=472.057) reflecting substantial variation in distribution mass. SMOTE also shows large variations. MAHAKIL yields lower EMD values (e.g. Potassium=5.13, Sulphate=9.70), indicating that the closer alignment with the original distribution.

In line with these values the KDE plots from Figure F1-F3shows that the real and synthetic data distributions of the features are nearly overlapping for few attributes. The KDE plots indicates that the generated synthetic data is slightly deviating from the original data points.

FigureF2: KDE Plot of Drinking Water Final Dataset oversampled using MAHAKIL.

FigureF3: KDE Plot of Drinking Water Final Dataset oversampled using GAN.

Table B3.KS Statistic and EMDscores of the oversampling techniques over Water Potability Dataset

|  | SMOTE | | | MAHAKIL | | | GAN | | |
| --- | --- | --- | --- | --- | --- | --- | --- | --- | --- |
| Feature | **KS Statistic** | **KS**  **p-value** | **EMD** | **KS**  **Statistic** | **KS**  **p-value** | **EMD** | **KS Statistic** | **KS**  **p-value** | **EMD** |
| ph | 0.1301 | 0.000 | 0.5105 | 0.121459 | 0.0000 | 0.44454 | 1.0000 | 0.000 | 6.6470 |
| Hardness | 0.0540 | 0.122 | 4.0618 | 0.068710 | 0.0219 | 4.80474 | 1.0000 | 0.000 | 191.1108 |
| Solids | 0.0233 | 0.953 | 279.95 | 0.068578 | 0.0223 | 1288.34 | 1.0000 | 0.000 | 22048.179 |
| Chloramines | 0.0584 | 0.075 | 0.2294 | 0.070521 | 0.0172 | 0.25606 | 0.9989 | 0.000 | 6.2058 |
| Sulfate | 0.0902 | 0.000 | 8.0215 | 0.185703 | 0.0000 | 19.9335 | 1.0000 | 0.000 | 280.0835 |
| Conductivity | 0.0826 | 0.003 | 12.392 | 0.063462 | 0.0424 | 10.5046 | 1.0000 | 0.000 | 423.7910 |
| Organic_carbon | 0.0809 | 0.003 | 0.5980 | 0.075244 | 0.0089 | 0.48623 | 1.0000 | 0.000 | 14.4502 |
| Trihalomethanes | 0.0639 | 0.040 | 3.3443 | 0.067364 | 0.0260 | 2.81327 | 1.0000 | 0.000 | 63.5229 |
| Turbidity | 0.0749 | 0.009 | 0.1342 | 0.086967 | 0.0014 | 0.13881 | 1.0000 | 0.000 | 3.9379 |

**Evaluation of Synthetic Water Potability Dataset:** From KS test results presented in TABLE B3, it is noticed that the SMOTE produces relatively low KS statistics for majority of the features (Solids=0.0233, Hardness=0.0540, and Chloramines=0.0584), with corresponding higher p-values (e.g. Solids=0.953, Hardness=0.122). Indicates that the SMOTE is able to reasonably preserve the original data distribution. MAHAKIL shows comparable but less consistent performance. In contrast, GAN provide extreme divergence from the original dataset, with KS statistic values are equal or near to 1.0 across all features and p-values of 0.00, which clearly indicating that complete distributional mismatch.

Figure F4. KDE Plot of Water potability Dataset oversampled using SMOTE.

Figure F5. KDE Plot of Water potability Dataset oversampled using MAHAKIL.

Figure F6: KDE Plot of Water potability Dataset oversampled using GAN.

Table B4 KS Statistic and EMD scores of the oversampling techniques over Water Quality Analysis Dataset

|  | **SMOTE** | | | **MAHAKIL** | | | **GAN** | | |
| --- | --- | --- | --- | --- | --- | --- | --- | --- | --- |
| Feature | **KS Statistic** | **KS**  **p-value** | **EMD** | **KS**  **Statistic** | **KS**  **p-value** | **EMD** | **KS Statistic** | **KS**  **p-value** | **EMD** |
| aluminium | 0.5458 | 0.0000 | 1.1352 | 0.481923 | 0.000000 | 0.563233 | 1.0000 | 0.0000 | 1.6331 |
| ammonia | 0.0484 | 0.0000 | 0.6283 | 0.102462 | 0.000000 | 1.723175 | 0.6678 | 0.0000 | 9.3939 |
| arsenic | 0.3189 | 0.0000 | 0.0882 | 0.097310 | 0.000000 | 0.042013 | 0.9551 | 0.0000 | 1.2924 |
| barium | 0.2070 | 0.0000 | 0.3795 | 0.152165 | 0.000000 | 0.252325 | 1.0000 | 0.0000 | 3.2745 |
| cadmium | 0.4423 | 0.0000 | 0.0263 | 0.210930 | 0.000000 | 0.012332 | 1.0000 | 0.0000 | 1.4433 |
| chloramine | 0.3808 | 0.0000 | 1.3410 | 0.324043 | 0.000000 | 0.774129 | 0.4433 | 0.0000 | 1.8938 |
| chromium | 0.3940 | 0.0000 | 0.1460 | 0.326611 | 0.000000 | 0.079739 | 0.7265 | 0.0000 | 0.6789 |
| copper | 0.1863 | 0.0000 | 0.1388 | 0.193015 | 0.000000 | 0.142405 | 0.6453 | 0.0000 | 0.7606 |
| flouride | 0.0997 | 0.0000 | 0.0846 | 0.095347 | 0.000000 | 0.078393 | 0.4959 | 0.0000 | 0.3718 |
| bacteria | 0.1815 | 0.0000 | 0.0621 | 0.223258 | 0.000000 | 0.065768 | 1.0000 | 0.0000 | 0.9414 |
| viruses | 0.2186 | 0.0000 | 0.1085 | 0.168911 | 0.000000 | 0.092616 | 0.9925 | 0.0000 | 1.5855 |
| lead | 0.1042 | 0.0000 | 0.0113 | 0.096186 | 0.000000 | 0.010528 | 1.0000 | 0.0000 | 0.9164 |
| nitrates | 0.1102 | 0.0000 | 1.1775 | 0.110227 | 0.000000 | 1.088986 | 0.6872 | 0.0000 | 6.2109 |
| nitrites | 0.1399 | 0.0000 | 0.1658 | 0.090698 | 0.000000 | 0.119035 | 0.2070 | 0.0000 | 0.2414 |
| mercury | 0.1502 | 0.0000 | 0.0005 | 0.141594 | 0.000000 | 0.000527 | 1.0000 | 0.0000 | 1.7376 |
| perchlorate | 0.2549 | 0.0000 | 5.7279 | 0.228088 | 0.000000 | 4.929024 | 0.4967 | 0.0000 | 13.4684 |
| radium | 0.1555 | 0.0000 | 0.4287 | 0.157045 | 0.000000 | 0.473161 | 0.3849 | 0.0000 | 1.4751 |
| selenium | 0.1316 | 0.0000 | 0.0051 | 0.128203 | 0.000000 | 0.005317 | 0.9953 | 0.0000 | 0.4242 |
| silver | 0.2393 | 0.0000 | 0.0439 | 0.199750 | 0.000000 | 0.030408 | 0.4302 | 0.0000 | 0.0738 |
| uranium | 0.2012 | 0.0000 | 0.0080 | 0.158757 | 0.000000 | 0.005703 | 1.0000 | 0.0000 | 1.1276 |
|  | | | | | | | | | |

Figure F7. KDE Plot of Water Quality Analysis Dataset oversampled using SMOTE.

**Evaluation of Synthetic Water Quality Analysis Dataset:** The SMOTE and MAHAKIL oversampling methods KS and EMD statistic values (TABLE B4) indicate that the all three oversampling methods yields p-values equal to 0.00 for most of the attributes, indicating the statistically significant difference between original and synthetic data distributions. Which in turn suggests that the none of the oversampling methods perfectly preserve the underlying data distribution for Water Quality Analysis Dataset.

The EMD values further support these observations. MAHAKIL generally achieve a low EMD values across most of the features (arsenic=0.0420, cadmium=0.0123, chromium=0.0797), indicating closer distributional proximity in terms of probability mass. SMOTE exhibits large EMD values for some features (e.g. perchlorate: 5.7279, aluminium: 1.1352), indicate larger deviations. Similarly GAN based oversampling method produces larger EMD values (e.g. ammonia=9.3939, percholorate=13.4684) indicating substantial distortion is generated in data.

Figure F8. KDE Plot of Water Quality Analysis Dataset oversampled using MAHAKIL.

Figure F9. KDE Plot of Water Quality Analysis Dataset oversampled using GAN.

3. **Correlation mapping**

Correlation map (Real, Synthetic and Difference) of Drinking Water Final oversampled dataset using SMOTE, MAHAKIL and GAN.

Figure F10.Correlation map (Real, Synthetic and Difference) of Drinking Water Final oversampled dataset using SMOTE

Figure F11.Correlation map (Real, Synthetic and Difference) of Drinking Water Final oversampled dataset using MAHAKIL

Figure F12.Correlation map (Real, Synthetic and Difference) of Drinking Water Final oversampled dataset using GAN

Correlation map (Real, Synthetic and Difference) of Waterpotability oversampled dataset using SMOTE, MAHAKIL and GAN

Figure F13.Correlation map (Real, Synthetic and Difference) of Water potability oversampled dataset using SMOTE

Figure F14.Correlation map (Real, Synthetic and Difference) of Water potability oversampled dataset using MAHAKIL

Figure F15.Correlation map (Real, Synthetic and Difference) of Water potability oversampled dataset using GAN

Correlation map (Real, Synthetic and Difference) of Water Quality Analysis oversampled dataset using SMOTE, MAHAKIL and GAN

Figure F16.Correlation map (Real, Synthetic and Difference) of Water Quality Analysisoversampled dataset using SMOTE

Figure F17.Correlation map (Real, Synthetic and Difference) of Water Quality Analysisoversampled dataset using MAHAKIL

Figure F18. Correlation map (Real, Synthetic and Difference) of Water Quality AnalysisOversampled dataset using GAN

4. **t-distributed stochastic neighbor embedding (t-SNE)**

The t-SNE visualizations provided a qualitative assessment of the degree to which the synthetic datasets preserved the structure of original water quality datasets. It is important to note that:

- Strong overlap between original and synthetic data points indicates good preservation of local data structure.
- Cluster misalignment or separation suggests that he synthetic data fails to capture the underlying distribution.
- Scattered or distorted clusters indicate poor convergence of the oversampling technique and potential loss of feature relationships.

|  |  |  |
| --- | --- | --- |
| (a) | (b) | (c) |

Figure F19. t-SNE map of Drinking Water Final oversampled dataset, a. t-SNE map of Drinking Water Final using SMOTE, b. t-SNE map of Drinking Water Final using MAHAKIL, c. t-SNE map of Drinking Water Final using GAN

|  |  |  |
| --- | --- | --- |
| (a) | (b) | (c) |

Figure F20. t-SNE map of Water Potability oversampled dataset, a. tt-SNE map of Water potability dataset using SMOTE, bt-SNE map of Water potability dataset using MAHAKIL, c. t-SNE map of Water potability dataset using GAN

|  |  |  |
| --- | --- | --- |
| (a) | (b) | (c) |

Figure F21. t-SNE map of Water Quality Analysis oversampled dataset, a. t-SNE map of Water Quality Analysis dataset using SMOTE, bt-SNE map of Water Quality Analysis dataset using MAHAKIL, c. t-SNE map of Water Quality Analysis dataset using GAN

**Drinking Water Final oversampled Dataset** (Figure 19) The t-SNE projections shows that the clusters formed by the synthetic datasets do not fully align with those of the original dataset.

SMOTE exhibits relatively better alignment compared to the other methods. MAHAKIL demonstrates noticeable cluster distortion, indicating moderate structural deviation. GAN produces more dispersed and less coherent clusters, highlighting that it fails to accurately learn the underlying data manifold.

**Water Potability oversampled dataset (Figure 20)** The visualizations present the similar trend where: The SMOTE provides comparatively better overlap between original and synthetic datasets. MAHAKIL shows partial overlap. GAN presents compactness compared to its performance on the Drinking Water Final oversampled dataset.

Water Quality Analysis Dataset (Figure 21) The t-SNE plots for this dataset show significant overlap between the original and synthetic datasets across the three oversampling methods. The SMOTE and GAN exhibit almost identical patterns and clustering overlap. The MAHAKIL oversampling method shows close alignment, with minor deviations.

From the above figures (F19-F21) the oversampling methods SMOTE, MAHAKIL and GAN performed well on the Water Quality Analysis dataset. The synthetic data generated is close and overlapping with the original data. Hence the synthetic data generated over the Water Quality Analysis dataset is appropriate for model training process.

**APPENDIX C**

To ensure robustness and mitigate model performance variability stemming from random initialization and sampling effects, all machine learning models—XGBoost (XGB), Random Forest (RF), Logistic Regression (LR), and Gradient Boosting (GB)—were assessed across many randomized experimental iterations. We used assessment metrics like Accuracy, Precision, Recall, F1-score, and ROC–AUC score to see how well the model worked for each run. We combined the findings from multiple runs and present the mean ± standard deviation (SD) for each statistic. The calculated values of the four models applied to the oversampled datasets are compiled and displayed in APPENDIX C. This method gives a more accurate picture of how well a model works and helps figure out if the differences across models are statistically significant.

TableC1. Classifiers Performance over Drinking water Final Dataset oversampled using SMOTE

| Metric | Accuracy | | Precision | | Recall | | F1-score | | Balanced  Accuracy | | AUC-PR | |
| --- | --- | --- | --- | --- | --- | --- | --- | --- | --- | --- | --- | --- |
| Model | mean | std | mean | std | mean | std | mean | std | mean | std | mean | std |
| GB | 0.995 | 0.01 | 0.997 | 0.01 | 0.99 | 0.01 | 0.99 | 0.01 | 0.995 | 0.01 | 0.997 | 0.0062 |
| LR | 0.995 | 0.01 | 0.995 | 0.01 | 0.99 | 0.01 | 0.99 | 0.01 | 0.995 | 0.01 | 0.999 | 0.0028 |
| RF | 0.999 | 0 | 0.997 | 0.01 | 1 | 0 | 1 | 0 | 0.999 | 0 | 1 | 0 |
| XGB | 0.997 | 0 | 0.995 | 0.01 | 1 | 0 | 1 | 0 | 0.998 | 0 | 1 | 0.0001 |

Table C2. Classifiers Performance over Drinking water Final Dataset oversampled using MAHAKIL

| Metric | Accuracy | | Precision | | Recall | | F1-score | | Balanced Accuracy | | AUC-PR | |
| --- | --- | --- | --- | --- | --- | --- | --- | --- | --- | --- | --- | --- |
| Model | mean | std | mean | std | mean | std | mean | std | mean | std | mean | std |
| GB | 0.99 | 0 | 1 | 0 | 0.988 | 0 | 1 | 0 | 0.994 | 0 | 1 | 0 |
| LR | 0.98 | 0 | 1 | 0.006 | 0.958 | 0 | 1 | 0 | 0.977 | 0 | 1 | 0 |
| RF | 1 | 0 | 1 | 0 | 0.998 | 0 | 1 | 0 | 0.999 | 0 | 1 | 0 |
| XGB | 1 | 0 | 1 | 0 | 1 | 0 | 1 | 0 | 1 | 0 | 1 | 0 |

Table C3. Classifiers Performance over Drinking water Final Dataset oversampled using GAN

| Metric | Accuracy | | Precision | | Recall | | F1-score | | Balanced Accuracy | | AUC-PR | |
| --- | --- | --- | --- | --- | --- | --- | --- | --- | --- | --- | --- | --- |
| Model | mean | std | mean | std | mean | std | mean | std | mean | std | mean | std |
| GB | 1.0 | 0 | 1 | 0 | 1 | 0 | 1 | 0 | 1 | 0 | 1 | 0 |
| LR | 1.0 | 0 | 1 | 0 | 1 | 0 | 1 | 0 | 1 | 0 | 1 | 0 |
| RF | 1.0 | 0 | 1 | 0 | 1 | 0 | 1 | 0 | 1 | 0 | 1 | 0 |
| XGB | 99.83 | 0.0038 | 0.9951 | 0.0109 | 1 | 0 | 0.9975 | 0.0055 | 0.9987 | 0.0029 | 0.9951 | 0.0109 |

Table C4. Classifiers Performance over Water potability Dataset oversampled using SMOTE

| Metric | Accuracy | | Precision | | Recall | | F1-score | | Balanced Accuracy | | AUC-PR | |
| --- | --- | --- | --- | --- | --- | --- | --- | --- | --- | --- | --- | --- |
| Model | mean | std | mean | std | mean | std | mean | std | mean | std | mean | std |
| GB | 0.616 | 0.011 | 0.623 | 0.025 | 0.618 | 0.014 | 0.62 | 0.01 | 0.617 | 0.011 | 0.687 | 0.021 |
| LR | 0.508 | 0.018 | 0.518 | 0.026 | 0.497 | 0.067 | 0.504 | 0.032 | 0.511 | 0.017 | 0.516 | 0.027 |
| RF | 0.685 | 0.008 | 0.702 | 0.018 | 0.660 | 0.020 | 0.68 | 0.007 | 0.686 | 0.008 | 0.779 | 0.021 |
| XGB | 0.64 | 0.028 | 0.647 | 0.034 | 0.637 | 0.029 | 0.641 | 0.03 | 0.64 | 0.028 | 0.709 | 0.031 |

Table C5. Classifiers Performance over Water potability Dataset oversampled using MAHAKIL

|  | Accuracy | | Precision | | Recall | | F1-score | | Balanced Accuracy | | AUC-PR | |
| --- | --- | --- | --- | --- | --- | --- | --- | --- | --- | --- | --- | --- |
|  | mean | std | mean | std | mean | std | mean | std | mean | std | mean | std |
| GB | 0.606 | 0.016 | 0.497 | 0.0209 | 0.511 | 0.016 | 0.504 | 0.018 | 0.589 | 0.016 | 0.526 | 0.009 |
| LR | 0.510 | 0.012 | 0.401 | 0.0126 | 0.516 | 0.018 | 0.451 | 0.014 | 0.511 | 0.013 | 0.421 | 0.008 |
| RF | 0.636 | 0.013 | 0.534 | 0.0171 | 0.537 | 0.024 | 0.535 | 0.017 | 0.618 | 0.013 | 0.55 | 0.012 |
| XGB | 0.626 | 0.013 | 0.519 | 0.0159 | 0.576 | 0.014 | 0.546 | 0.013 | 0.617 | 0.012 | 0.558 | 0.011 |

Table C6. Classifiers Performance over Water potability Dataset oversampled using GAN

| Metric | Accuracy | | Precision | | Recall | | F1-score | | Balanced Accuracy | | AUC-PR | |
| --- | --- | --- | --- | --- | --- | --- | --- | --- | --- | --- | --- | --- |
| Model | mean | std | mean | std | mean | std | mean | std | mean | std | mean | std |
| GB | 0.639 | 0.001 | 0.583 | 0.003 | 0.269 | 0.002 | 0.3687 | 0.003 | 0.573 | 0.001 | 0.657 | 0.001 |
| LR | 0.596 | 0 | 0.442 | 0 | 0.121 | 0 | 0.1901 | 0 | 0.511 | 0 | 0.499 | 0 |
| RF | 0.671 | 0.009 | 0.641 | 0.021 | 0.360 | 0.014 | 0.4614 | 0.017 | 0.615 | 0.010 | 0.681 | 0.007 |
| XGB | 0.627 | 0 | 0.529 | 0 | 0.417 | 0 | 0.4672 | 0 | 0.589 | 0 | 0.654 | 0 |

Table C7. Classifiers Performance over Water Quality Analysis Dataset oversampled using SMOTE

|  | Accuracy | | Precision | | Recall | | F1-score | | Balanced Accuracy | | AUC-PR | |
| --- | --- | --- | --- | --- | --- | --- | --- | --- | --- | --- | --- | --- |
|  | mean | std | mean | std | mean | std | mean | std | mean | std | mean | std |
| GB | 0.957 | 0.002 | 0.95 | 0.007 | 0.9662 | 0.009 | 0.958 | 0.002 | 0.958 | 0.002 | 0.993 | 0 |
| LR | 0.801 | 0.004 | 0.819 | 0.008 | 0.773 | 0.003 | 0.795 | 0.003 | 0.801 | 0.004 | 0.861 | 0.01 |
| RF | 0.975 | 0.004 | 0.97 | 0.008 | 0.9791 | 0.005 | 0.975 | 0.004 | 0.975 | 0.004 | 0.998 | 0 |
| XGB | 0.982 | 0.002 | 0.978 | 0.004 | 0.9857 | 0.002 | 0.982 | 0.002 | 0.982 | 0.002 | 0.999 | 0 |

Table C8. Classifiers Performance over Water Quality Analysis Dataset oversampled using MAHAKIL

|  | Accuracy | | Precision | | Recall | | F1-score | | Balanced Accuracy | | AUC-PR | |
| --- | --- | --- | --- | --- | --- | --- | --- | --- | --- | --- | --- | --- |
|  | mean | std | mean | std | mean | std | mean | std | mean | std | mean | std |
| GB | 0.917 | 0.003 | 0.6194 | 0.013 | 0.716 | 0.004 | 0.664 | 0.008 | 0.829 | 0.002 | 0.736 | 0.007 |
| LR | 0.747 | 0.004 | 0.2826 | 0.003 | 0.791 | 0.003 | 0.416 | 0.003 | 0.766 | 0.002 | 0.516 | 0.002 |
| RF | 0.922 | 0.001 | 0.6376 | 0.005 | 0.730 | 0.012 | 0.680 | 0.005 | 0.8387 | 0.005 | 0.718 | 0.009 |
| XGB | 0.960 | 0.003 | 0.8764 | 0.022 | 0.762 | 0.017 | 0.815 | 0.018 | 0.8744 | 0.009 | 0.889 | 0.008 |

Table C9. Classifiers Performance over Water Quality Analysis Dataset oversampled using GAN

|  | Accuracy | | Precision | | Recall | | F1-score | | Balanced Accuracy | | AUC-PR | |
| --- | --- | --- | --- | --- | --- | --- | --- | --- | --- | --- | --- | --- |
|  | mean | std | mean | std | mean | std | mean | std | mean | std | mean | std |
| GB | 0.956 | 0 | 0.911 | 0 | 0.681 | 0 | 0.779 | 0 | 0.836 | 0 | 0.978 | 0 |
| LR | 0.908 | 0 | 0.716 | 0 | 0.318 | 0 | 0.441 | 0 | 0.651 | 0 | 0.830 | 0 |
| RF | 0.960 | 0.001 | 0.940 | 0.014 | 0.695 | 0.008 | 0.799 | 0.008 | 0.844 | 0.004 | 0.973 | 0.002 |
| XGB | 0.971 | 0 | 0.930 | 0 | 0.807 | 0 | 0.864 | 0 | 0.899 | 0 | 0.985 | 0 |

**APPENDIX D**

The following Table D1 provides the details about the hyperparameters for each model, along with the selected values across 5 random seed values.

Table D1. Hyperparameters optimal values for the classifier

| **Model** | **Hyperparameter** | **Optimal Value** |
| --- | --- | --- |
| RF | n_estimators | 100 |
|  | Max_depth | None |
|  | random_state | [23, 42, 212, 401, 500] |
|  | Criterion | gini |
|  | min_samples_leaf | 1 |
|  | max_features | sqrt |
|  | appha | 0.0 |
| LR | Iterations | 1000 |
|  | Penalty | L2 |
|  | random_state | [23, 42, 212, 401, 500] |
|  | verbose | 0 |
| XGB | eval_metric | Logloss |
|  | max_depth' | 6 |
|  | learning_rate' | 0.3 |
|  | no_iterations | 783 |
|  | no_estimators | 100 |
|  | gamma | 0 |
|  | alpha | 0 |
|  | random_state | [23, 42, 212, 401, 500] |
| GBM | no_estimators | 685 |
|  | max_depth | 13 |
|  | l_rate | 0.01 |
|  | random_state | [23, 42, 212, 401, 500] |
|  | min_sample_split | 2 |
|  | sub_sample | 1.0 |
|  | Max_depth | 3 |
|  | appha | 0.0 |

**APPENDIX E**

In addition to the standard accuracy metric, we have applied multiple (class-wise) metrics other metrics to assess the performance of models, the metrics include Precision–Recall AUC (AUPRC), Matthews Correlation Coefficient (MCC), Class-wise precision, recall, and F1-score, and Calibration plots and Expected Calibration Error (ECE).

Table E1: The LR, RF, XGB and GB models class-wise precision, recall and f1 values over Drinking Water Final Dataset oversampled using SMOTE

| **Model** | **CLASS 0** | | | **CLASS 1** | | |
| --- | --- | --- | --- | --- | --- | --- |
|  | **precision** | **recall** | **f1-score** | **precision** | **recall** | **f1-score** |
| LR | 0.997368 | 0.984416 | 0.990832 | 0.98468 | 0.997403 | 0.990984 |
| RF | 1 | 0.997403 | 0.998693 | 0.997436 | 1 | 0.99871 |
| XGB | 1 | 0.992208 | 0.996078 | 0.992308 | 1 | 0.996129 |
| GB | 0.994872 | 0.997403 | 0.996129 | 0.997368 | 0.994805 | 0.996078 |

Table E2: The LR, RF, XGB and GB models class-wise values of AUPRC, MCC & ECE over Drinking Water Final Dataset oversampled using SMOTE

| **Model** | **AUPRC** | **MCC** | **ECE** |
| --- | --- | --- | --- |
| LR | 0.99807 | 0.981933 | 0.023466 |
| RF | 1 | 0.997419 | 0.022727 |
| XGB | 0.99929 | 0.992258 | 0.004568 |
| GB | 0.994839 | 0.992224 | 0.003909 |

Table E3: The LR, RF, XGB and GB models class-wise precision, recall and f1 values over Drinking Water Final Dataset oversampled using MAHAKIL

| **Model** | **CLASS 0** | | | **CLASS 1** | | |
| --- | --- | --- | --- | --- | --- | --- |
|  | **precision** | **recall** | **f1-score** | **precision** | **recall** | **f1-score** |
| LR | 0.992405 | 0.984416 | 0.988301 | 0.984643 | 0.992208 | 0.988318 |
| RF | 0.997403 | 0.994805 | 0.996095 | 0.994838 | 0.997403 | 0.996112 |
| XGB | 0.997403 | 0.992208 | 0.994788 | 0.992274 | 0.997403 | 0.994822 |
| GB | 0.997403 | 0.994805 | 0.996095 | 0.994838 | 0.997403 | 0.996112 |

Table E4: The LR, RF, XGB and GB models class-wise values of AUPRC, MCC & ECE over Drinking Water Final Dataset oversampled using MAHAKIL

| **Model** | **AUPRC** | **MCC** | **ECE** |
| --- | --- | --- | --- |
| LR | 0.995361 | 0.976836 | 0.022263 |
| RF | 0.999864 | 0.992224 | 0.022234 |
| XGB | 0.99899 | 0.989644 | 0.006411 |
| GB | 0.993574 | 0.992224 | 0.004393 |

Table E5: The LR, RF, XGB and GB models class-wise precision, recall and f1 values over Drinking Water Final Dataset oversampled using GAN

| **Model** | **CLASS 0** | | | **CLASS 1** | | |
| --- | --- | --- | --- | --- | --- | --- |
|  | **precision** | **recall** | **f1-score** | **precision** | **recall** | **f1-score** |
| LR | 1 | 1 | 1 | 1 | 1 | 1 |
| RF | 1 | 1 | 1 | 1 | 1 | 1 |
| XGB | 1 | 1 | 1 | 1 | 1 | 1 |
| GB | 1 | 1 | 1 | 1 | 1 | 1 |

Table E6: The LR, RG, XGB and GB models class-wise values of AUPRC, MCC & ECE over Drinking Water Final Dataset oversampled using GAN

| **Model** | **AUPRC** | **MCC** | **ECE** |
| --- | --- | --- | --- |
| LR | 1 | 1 | 0.000879 |
| RF | 1 | 1 | 0.002427 |
| XGB | 1 | 1 | 0.003801 |
| GB | 1 | 1 | 0.00046 |

From Table(s) E1 to Table E6 representing the LR, RF, XGB and GB models class wise-precision, recall and f1-scores. Tables representing the AUPRC, MCC over the Drinking Water Final Dataset using three different oversampling methods SMOTE, MAHAKIL and GAN respectively. From the Table(s) E1, E3and Table E5 it is noticed the LR, RF, GB and XGB models performed well on the over Drinking Water Final Dataset oversampled using GAN with 100% precision, recall and f1-score. The MCC of all these models attained a value of 1 indicating the prefect prediction among potable and non-potable classes.

From Table(s) E2, E4 and Table E6 all the four models (LR, RG, XGB and GB) attained near perfect AUPRC values (>0.99), indicating excellent ability to discriminate potable and non-potable cases. Overall, the SMOTE produces highly separable data, making all the four models to perform well.

Table E7: The LR, RG, XGB and GB models class-wise precision, recall and f1 values over Water potability Dataset oversampled using SMOTE

| **Model** | **CLASS 0** | | | **CLASS 1** | | |
| --- | --- | --- | --- | --- | --- | --- |
|  | **precision** | **recall** | **f1-score** | **precision** | **recall** | **f1-score** |
| LR | 0.512665 | 0.49 | 0.500703 | 0.511258 | 0.53375 | 0.521939 |
| RF | 0.677102 | 0.7025 | 0.689481 | 0.690456 | 0.664375 | 0.677069 |
| XGB | 0.652404 | 0.6375 | 0.644832 | 0.645328 | 0.66 | 0.652551 |
| GB | 0.62813 | 0.621875 | 0.62485 | 0.625866 | 0.631875 | 0.62872 |

Table E8: The LR, RG, XGB and GB models class-wise AUPRC, MCC & ECE values over Water potability Dataset oversampled using SMOTE

| **Model** | **AUPRC** | **MCC** | **ECE** |
| --- | --- | --- | --- |
| LR | 0.519794 | 0.023836 | 0.021947 |
| RF | 0.77106 | **0.367216** | 0.075934 |
| XGB | 0.712224 | 0.297616 | 0.125615 |
| GB | 0.672922 | 0.253873 | 0.04784 |

Table E9: The LR, RG, XGB and GB models class-wise precision, recall and f1 values over Water potability Dataset oversampled using MAHAKIL

| **Model** | **CLASS 0** | | | **CLASS 1** | | |
| --- | --- | --- | --- | --- | --- | --- |
|  | **precision** | **recall** | **f1-score** | **precision** | **recall** | **f1-score** |
| LR | 0.504319 | 0.51 | 0.507074 | 0.503809 | 0.498125 | 0.500881 |
| RF | 0.612986 | 0.665 | 0.637859 | 0.633939 | 0.58 | 0.605667 |
| XGB | 0.589783 | 0.60625 | 0.597714 | 0.595616 | 0.57875 | 0.586864 |
| GB | 0.604103 | 0.6175 | 0.610545 | 0.609424 | 0.595625 | 0.602247 |

Table E10: The LR, RG, XGB and GB models class-wise AUPRC, MCC & ECE values over Water potability Dataset oversampled using MAHAKIL

| **Model** | **AUPRC** | **MCC** | **ECE** |
| --- | --- | --- | --- |
| LR | 0.512577 | 0.008126 | 0.026642 |
| RF | 0.689902 | **0.24596** | 0.037847 |
| XGB | 0.663975 | 0.185199 | 0.165188 |
| GB | 0.670937 | 0.213326 | 0.035336 |

Table E11: The LR, RG, XGB and GB models class-wise precision, recall and f1 values over Water potability Dataset oversampled using GAN

| **Model** | **CLASS 0** | | | **CLASS 1** | | |
| --- | --- | --- | --- | --- | --- | --- |
|  | **precision** | **recall** | **f1-score** | **precision** | **recall** | **f1-score** |
| LR | 0.603015 | 0.902256 | 0.722892 | 0.327586 | 0.074219 | 0.121019 |
| RF | 0.678305 | 0.869674 | 0.762132 | 0.637454 | 0.357031 | 0.457506 |
| XGB | 0.694064 | 0.761905 | 0.726404 | 0.562212 | 0.476563 | 0.515856 |
| GB | 0.651493 | 0.875188 | 0.746952 | 0.581515 | 0.270313 | 0.369065 |

Table E12: The LR, RG, XGB and GB models class-wise AUPRC, MCC & ECE values over Water potability Dataset oversampled using GAN

| **Model** | **AUPRC** | **MCC** | **ECE** |
| --- | --- | --- | --- |
| LR | 0.50375 | 0.04041 | 0.036599 |
| RF | 0.6828 | **0.26752** | 0.032061 |
| XGB | 0.65859 | 0.247211 | 0.133468 |
| GB | 0.668079 | 0.184127 | 0.040067 |

From table(s) E7-E12 representing the LR, RF, XGB and GB models class wise-precision, recall and f1-scores. Tables representing the AUPRC, MCC over the Water potability Dataset using three different oversampling methods SMOTE, MAHAKIL and GAN respectively. From the Table(s) E7, E9 and Table E11 it is noticed the RF model attain the best performance across both classes, showing balanced precision and recall. The LR model is the weakest, confirming limited model capacity over the Water potability Dataset. The LR model performance with respective to GAN oversampling shows a string bias towards Class-0, with very high recall (0.902) indicating overfitting or model collapse towards the majority distribution. The GAN based oversampling improves majority class performance, it fails to capture minority class distribution.

From table(s) E8, E10 and Table E12, the three oversampling techniques, the AUPRC values indicate that the RF model consistently achieves the good tradeoff between precision-recall, while LR model close to the RF. Overall, the SMOTE provides the highest AUPRC values, followed by the MAHAKIL and GAN oversampling methods.

Table E13: The LR, RG, XGB and GB models class-wise precision, recall and f1 values over Water Quality Analysis Dataset oversampled using SMOTE

| **Model** | **CLASS 0** | | | **CLASS 1** | | |
| --- | --- | --- | --- | --- | --- | --- |
|  | **precision** | **recall** | **f1-score** | **precision** | **recall** | **f1-score** |
| LR | 0.788332 | 0.828748 | 0.808029 | 0.819454 | 0.777425 | 0.797879 |
| RF | 0.980947 | 0.971076 | 0.975984 | 0.971364 | 0.981129 | 0.97622 |
| XGB | **0.988037** | 0.975661 | 0.981804 | **0.975984** | 0.988183 | **0.982039** |
| GB | 0.968311 | 0.953616 | 0.960904 | 0.95431 | 0.968783 | 0.96149 |

Table E14: The LR, RG, XGB and GB models class-wise AUPRC, MCC & ECE values over Water Quality Analysis Dataset oversampled using SMOTE

| **Model** | **AUPRC** | **MCC** | **ECE** |
| --- | --- | --- | --- |
| LR | 0.857188 | 0.606979 | 0.054979 |
| RF | 0.99729 | 0.952258 | 0.05753 |
| XGB | 0.998715 | **0.963933** | 0.0059 |
| GB | 0.992734 | 0.92251 | 0.056906 |

Table E15: The LR, RG, XGB and GB models class-wise precision, recall and f1 values over Water Quality Analysis Dataset oversampled using MAHAKIL

| **Model** | **CLASS 0** | | | **CLASS 1** | | |
| --- | --- | --- | --- | --- | --- | --- |
|  | **precision** | **recall** | **f1-score** | **precision** | **recall** | **f1-score** |
| LR | 0.774245 | 0.83351 | 0.802717 | 0.81978 | 0.75679 | 0.786937 |
| RF | 0.980662 | 0.965961 | 0.973253 | 0.966475 | 0.980952 | 0.973657 |
| XGB | **0.987658** | 0.973545 | 0.980546 | **0.973932** | 0.987831 | **0.980827** |
| GB | 0.970643 | 0.949735 | 0.960064 | 0.950812 | 0.971252 | 0.960913 |

Table E16: The LR, RG, XGB and GB models class-wise AUPRC, MCC & ECE values over Water Quality Analysis Dataset oversampled using MAHAKIL

| **Model** | **AUPRC** | **MCC** | **ECE** |
| --- | --- | --- | --- |
| LR | 0.85087 | 0.592159 | 0.060611 |
| RF | 0.996407 | 0.947026 | 0.06308 |
| XGB | 0.998513 | **0.961483** | 0.008576 |
| GB | 0.991052 | 0.921221 | 0.058775 |

Table E17: The LR, RG, XGB and GB models class-wise precision, recall and f1 values over Water Quality Analysis Dataset oversampled using GAN

| **Model** | **CLASS 0** | | | **CLASS 1** | | |
| --- | --- | --- | --- | --- | --- | --- |
|  | **precision** | **recall** | **f1-score** | **precision** | **recall** | **f1-score** |
| LR | 0.911995 | 0.986601 | 0.947832 | 0.712121 | 0.258242 | 0.379032 |
| RF | 0.961527 | 0.993935 | 0.977461 | 0.936072 | 0.69011 | 0.794342 |
| XGB | **0.974412** | 0.993653 | **0.983939** | **0.941558** | 0.796703 | **0.863095** |
| GB | 0.952594 | 0.991961 | 0.971879 | 0.907632 | 0.615385 | 0.733466 |

Table E18: The LR, RG, XGB and GB models class-wise AUPRC, MCC & ECE values over Water Quality Analysis Dataset oversampled using GAN

| **Model** | **AUPRC** | **MCC** | **ECE** |
| --- | --- | --- | --- |
| LR | 0.821913 | 0.390909 | 0.013313 |
| RF | 0.973365 | 0.783519 | 0.036869 |
| XGB | 0.982447 | **0.850849** | 0.017165 |
| GB | 0.976638 | 0.722808 | 0.031186 |

Table(s) E13to E18 representing the LR, RF, XGB and GB models class wise-precision, recall and f1-scores. Tables representing the AUPRC, MCC over the Water Quality Analysis Dataset using three different oversampling methods SMOTE, MAHAKIL and GAN respectively. From the Table(s) E13, E15 and Table E17 it is noticed the XGB model attain the highest f1-score across three oversampling methods (SMOTE, MAHAKIL and GAN). The XGB model MCC score lies between 0.85-0.96 across the oversampling methods.

From Table(s) E14, E16 and Table E18 the AUPRC analysis revealed that SMOTE and MAHAKIL oversampling methods will provide the models with near perfect precision-recall tradeoff for the four models, with XGB consistently achieving highest performance. In contrast GAN based oversampling method results in comparatively lower AUCPR and MCC values. These findings suggest that GAN will be used to provide a reasonable oversampled dataset but it does not improve the classification effectiveness for the water quality analysis dataset.

**Calibration Analysis:**

Further, to assess the performance of the considered three oversampling techniques (SMOTE, MAHAKIL and GAN) generated calibration plots. The x-axis of the calibration plot denotes the mean predicted probability and y-axis denotes the probability of fraction of positives. If the curves near to the diagonal line indicates the model is well calibrated, if the curve is below the diagonal line means the predicted probabilities too high and if the curve is above the diagonal line, then the model predicts the probabilities too low.

|  |  |  |
| --- | --- | --- |
| a. SMOTE-Based Classifier Calibration | b. MAHAKIL-Based Classifier Calibration | C. GAN- Based Classifier Calibration |

**Figure F22:** Calibration Plots of Classifiers on Drinking Water Quality Final Dataset under Different Augmentation Strategies: (a) SMOTE, (b) MAHAKIL, and (c) GAN

**Classifier calibration on SMOTE-Augmented Drinking Water Quality Final Dataset**: From Figure 22 (a) it is noticed that XGB & GB model very close to the diagonal line and it is well calibrated. The RF model slightly deviated in the mid-range indicates mildly mis calibrated. Logistic regression model having poor calibration spread. STOME produces balanced synthetic samples leading to stable calibration for boosting models.

**Classifier calibration on MAHAKIL-Augmented Drinking Water Quality Final Dataset**: All the four models are deviated from the diagonal line and indicates poor calibration despite of high accuracy (Figure 22(b)).

**Classifier calibration on GAN-Augmented Drinking Water Quality Final Dataset**: As show in Figure 22 (c) the XGB & GB model very close to the diagonal line and it is well calibrated. The RF model shows under confidence. Logistic regression model is unstable. GAN generates more realistic synthetic samples

|  |  |  |
| --- | --- | --- |
| a. SMOTE-Based Classifier Calibration | b. MAHAKIL-Based Classifier Calibration | c. GAN- Based Classifier Calibration |

**Figure F23:** Calibration Plots of Classifiers on Water portability Dataset under Different Augmentation Strategies: (a) SMOTE, (b) MAHAKIL, and (c) GAN

As show in the Figure(s) 23 (a)- 23 (c) the calibration analysis indicates that models trained with STOME-based oversampling demonstrates good calibration, closely aligned with the reliability curve. The four classifiers trained with the MAHAKIL-oversampled data are underconfident. These findings indicate the MAHAKIL based oversampling will provide the class balance but adversely affect the probability estimation. Similarly, the RF & GB classifiers trained with the GAN based oversampled Water potability dataset are well calibrated and nearly aligns with the reliability curve. The XGB and LR models are underconfident.

|  |  |  |
| --- | --- | --- |
| a. SMOTE-Based Classifier Calibration | b. MAHAKIL-Based Classifier Calibration | C. GAN- Based Classifier Calibration |

**Figure F24**: Calibration Plots of Classifiers on Water Quality Analysis Dataset under Different Augmentation Strategies: (a) SMOTE, (b) MAHAKIL, and (c) GAN

The calibration analysis indicates that models trained with Water Quality Analysis Dataset oversampled with STOME demonstrates acceptable calibration and some deviations noticed in mid and high probability regions (Figure(s) 24(a)-24(c)). In contrast, the model’s calibration analysis with Water Quality Analysis Dataset oversampled with MAHAKIL are mostly underconfident and shows miscalibration across the probability bins. The calibration analysis with the GAN based Water quality analysis shows that the XGB, LR model provides unstable calibration. The RF and GB models showed slight overestimation at high probabilities. Overall, the RF and GB models remain well calibrated.
